# Recent advances in surface modification of micro- and nano-scale biomaterials with biological membranes and biomolecules

**DOI:** 10.3389/fbioe.2022.972790

**Published:** 2022-10-12

**Authors:** Manisha Sandupama Abesekara, Ying Chau

**Affiliations:** Department of Chemical and Biological Engineering, The Hong Kong University of Science and Technology, Kowloon, Hong Kong SAR, China

**Keywords:** biomaterials, surface modification, cell membranes, exosomes, biological membrane, microparticles, nanoparticles, particle coating

## Abstract

Surface modification of biomaterial can improve its biocompatibility and add new biofunctions, such as targeting specific tissues, communication with cells, and modulation of intracellular trafficking. Here, we summarize the use of various natural materials, namely, cell membrane, exosomes, proteins, peptides, lipids, fatty acids, and polysaccharides as coating materials on micron- and nano-sized particles and droplets with the functions imparted by coating with different materials. We discuss the applicability, operational parameters, and limitation of different coating techniques, from the more conventional approaches such as extrusion and sonication to the latest innovation seen on the microfluidics platform. Methods commonly used in the field to examine the coating, including its composition, physical dimension, stability, fluidity, permeability, and biological functions, are reviewed.

## Introduction

Biomaterial is defined as “a material that has been engineered to take a form which, alone or as part of a complex system, is used to direct, by control of interactions with components of living systems, the course of any therapeutic or diagnostic procedures” (Williams and David, 2014). Micro (0.5–1,000 µm)- and nano (<0.5 µm)-scale biomaterials allow mimicking natural cellular interactions with varying degrees of complexity (Balmert and Little, 2012). Over the past few decades, they have been extensively investigated for various medicinal purposes including drug delivery and cell therapy. In the current research, biomaterial does not merely serve as a structural support but also has an important role in engaging in active communication with the living system (Liu et al., 2020; Zeng et al., 2021). For example, Chung and coworkers found that the hydrogel matrix could amplify or suppress actions of cytokine signals in addition to being a control release depot or biological scaffold (Chung et al., 2021). The surface of biomaterial, which is in direct contact with the external environment, is, therefore, a major design factor in constructing a new biomaterial.

Surface modification or coating changes the physical, chemical, and biological properties of surfaces to improve the functionality of the bulk material. It is not simply attaching or coating with an insert material to cover the foreign biomaterial. A thorough and rational design considering molecular biology, reaction kinetics, and thermodynamics is needed to produce a realistic, stable, and functional interface. Prolonged circulation, enhanced biocompatibility, improved colloidal stability, and targeted delivery are some general advantages associated with surface modification (Chen et al., 2016; Zhu et al., 2018a; Wu et al., 2020; Ai et al., 2021).

**FIGURE 1:**
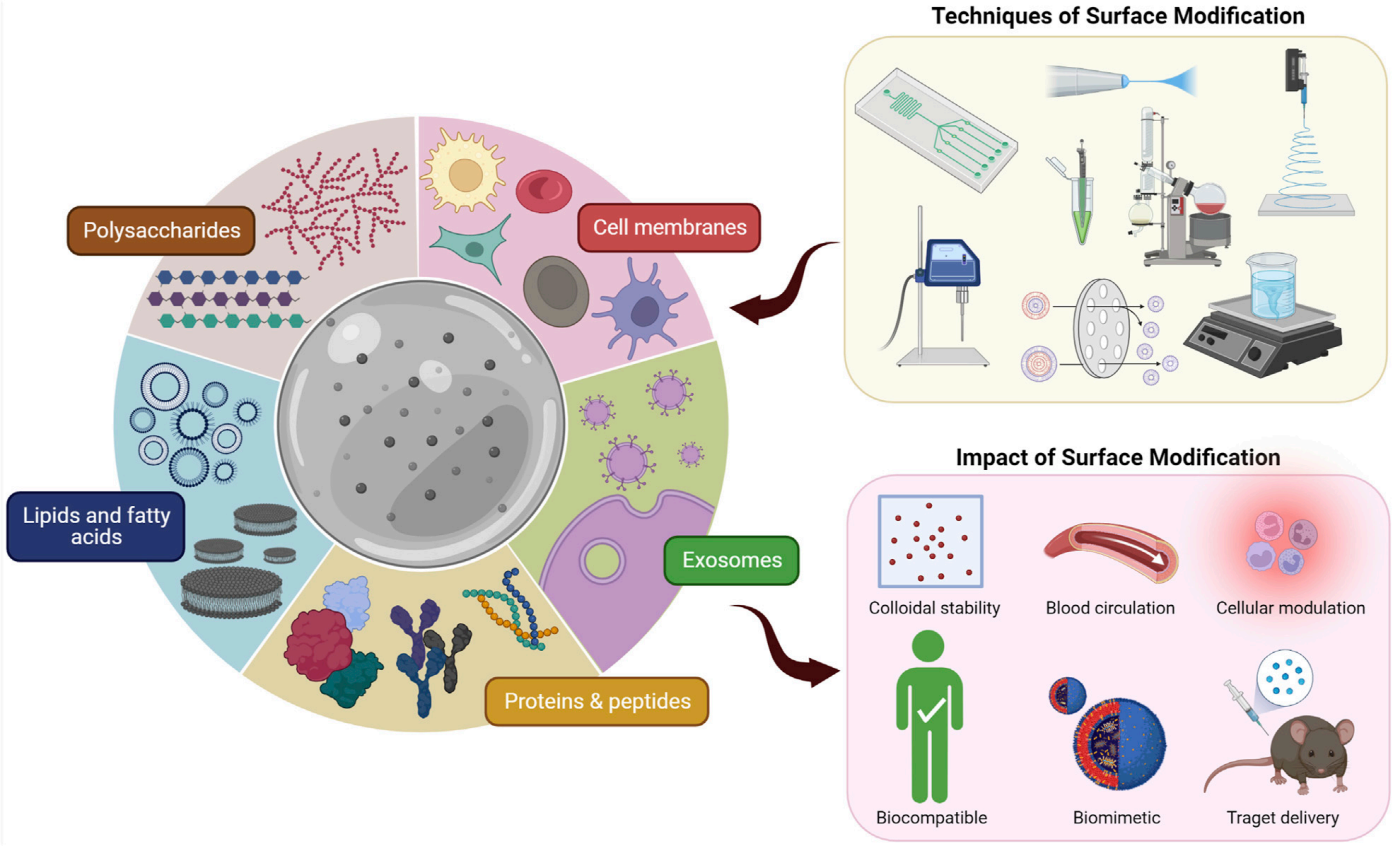
Different techniques of surface modification of micron- and nano-scale biomaterials using biological membranes and biomolecules and the impact thereof.

For the surface modification of micron- and nano-sized biomaterials, a wide range of materials covering both natural and synthetic materials have been successfully assessed. However, evidence on negative effects of synthetic materials as anti-PEG antibodies became a concern (Shiraishi and Yokoyama, 2019). Hence, investigations to find less immunogenic alternatives for synthetic materials have started (Hoang Thi et al., 2020). On the other hand, the research community has taken a keen interest in natural materials for surface modification of biomaterials to overcome the shortcomings of synthetic materials (Fang et al., 2014; Xu C. H. et al., 2020; Oroojalian et al., 2021). Consequently, biological membranes and biomolecules have been studied to modify micron- and nano-scale biomaterials’ surfaces (Table 1). We will explore the different strategies that have been used to perform surface modification of micron- and nano-scale biomaterials using biological membranes and biomolecules (Figure 1). Finally, we will discuss how to verify and characterize the surface coating.

**TABLE 1:** Advantages and disadvantages of biological membranes and biomolecules of surface modification.

Material	Advantage	Disadvantage
Cell membranes (Guo et al., 2021; Gao and Xiao, 2022)	Homotypic targeting	Less control over composition
	Colloidal stability	
Exosomes (Kang et al., 2022; Salarpour et al., 2022)	Homotypic targeting	Low supply
	Colloidal stability	Heterogeneity
Protein and peptides (Huang, et al., 2013; Li, Liu and Zhao, 2022)	Environmental responsive	Prone to denature
	Ease of customized design	
	Perform enzyme-mediated reactions	
Lipids and fatty acids (Krishnamurthy et al., 2015; Luchini and Vitiello, 2019)	Mimic cell membrane	Low colloidal stability
	Abundant supply	
	Amphiphilicity	
Polysaccharides (Shen et al., 2019; Truong et al., 2022)	Abundant supply	Material variability
	Diverse functional groups	Toxic contamination
	Thermal stability	

## Biological membranes and biomolecules of surface modification

Lipids are the most studied and oldest biomolecules of surface coating to mimic the cell membrane (Lombardo and Kiselev, 2022). Then, proteins and peptides have been widely used as a surface decoration to target and communicate with cells. Currently, some researchers are exploring the potential of proteins as a responsive surface coating (Huang et al., 2014b; Mu et al., 2021). For example, Huang et al. used protein–polymer nanoconjugates to construct a temperature-responsive semipermeable membrane around water-in-oil emulsion droplets (Huang et al., 2013). Polysaccharides have been used extensively to coat polymeric and metallic particles (Mincheva et al., 2008; Tang et al., 2016). Being a natural polymer, polysaccharides can undergo harsh chemical and physical (thermal and electrical) processes. Biological membranes include plasma membranes, intracellular membranes, and cell-derived membranes such as extracellular vesicle membranes. In recent years, the research community started to explore cell membranes and exosome membranes in surface modification (Gangadaran and Ahn, 2020; Zeng and Pu, 2020; Yu et al., 2022). Even though the cell membranes and exosomes are rich with targeting ligands, immune evasive ligands, and self-markers, the control over ligand expression and troubleshooting can be complex compared to previously mentioned biomolecules. This section will discuss types and characteristics of different biological membranes and biomolecules that can be applied in surface modification of micro- and nano-scale biomaterials.

### Cell membranes

The cell, the fundamental unit of life, is already equipped with a sophisticated plasma membrane to interact with the surrounding environment to perform a variety of complex functions such as identification, communication, and selective transportation. Although surface modification of biomaterials using components of the plasma membrane such as cell receptors is popular (Negahdaripour et al., 2017; Rhodes and Green, 2018; Ravasco et al., 2019), direct use of cell membranes (CMs) could be another appealing approach to preserve native functional components of original CMs. The direct use of CM saves researchers from labor-intensive proteomics and long multivalent surface functionalization processes. Consequently, CM coating has been investigated for potential to improve targeting and colloidal stability of micron- and nano-size biomaterials. Table 2 contains some CM types commonly used in surface modification of biomaterials. To combine different functions of different CMs, hybrid CMs can be prepared by coextrusion of multiple CMs (Chen et al., 2020). Additionally, CMs can be functionalized to express noninherent molecules by lipid insertion and chemical conjugation (Fang et al., 2013; Zhang et al., 2017; Li P. Y. et al, 2018; Chai et al., 2019; Ai et al., 2021).The intracellular membrane has a structural composition (lipids and proteins) similar to the CM. However, they are less diverse, thinner, and fragile, unlike the CM which is a robust functional barrier (Stewart, Langer and Jensen, 2018). Zhang et al. demonstrated the surface modification of poly (lactic-co-glycolic acid) (PLGA) nanoparticles (NPs) (∼103 nm) and single-walled carbon nanotube field-effect transistors with the outer mitochondrial membrane as a model intracellular membrane (Gong et al., 2021a). This shows the promising opportunity for intracellular membrane-coated biomaterials in future.

**TABLE 2:** Characteristics conferred from different CMs and their applications.

Cell membrane type	Characteristics conferred from CM coating	Application	Reference
Red blood cell	Prolonged blood circulation	Tumor imaging	(Aryal et al., 2013; Hu et al., 2013; Jiang et al., 2017; Rao et al., 2017; Xia et al., 2019)
	Tumor homing	Cancer therapy	
Macrophage	Accumulates in inflammatory sites	Cancer therapy	Cao et al. (2016), Cao et al. (2020), Choo et al. (2018); Zhang et al. (2020); Han et al. (2021); Gong et al. (2021b); Tang et al. (2021)
	Penetrates blood–brain barrier	Nano-sponges for virus	
	Polarizes macrophages (polarized macrophage CM)	Drug delivery for neurological diseases	
Dendritic cell	T-cell activation (mature dendritic CM)	Cancer therapy	(Liu et al., 2019b; Cheng et al., 2020)
	Tumor homing		
Natural-killer cell	Polarizes macrophages	Photodynamic therapy	Deng et al. (2018)
	Tumor homing		
T-cell	Tumor homing	Photothermal therapy	(Han et al., 2019; Yaman et al., 2020)
		Chemotherapy	
Platelets	Adhesiveness	Drug delivery	(Li et al., 2016; Wei et al., 2016)
		Detoxification	
Mesenchymal stem cell	Targets tumors at different developmental stages	Chemotherapy	(Toledano Furman et al., 2013; Gao et al., 2016; Liang et al., 2018; Wang et al., 2021)
	Accumulates in inflammatory sites	Anti-inflammatory drug delivery	
	Contains diverse set of receptors	Tissue regeneration	
Cancer cell	Tumor-specific antigen presentation	Cancer therapy	(Fang et al., 2014; Chen et al., 2016; Zhu et al., 2016; Sun et al., 2020; Wang et al., 2020; Zhao et al., 2020; Xiong et al., 2021)
	Homotypic binding	Tumor imaging	
	Reduced blood clearance	Gene therapy	
Outer mitochondrial membrane	Binding with membrane-specific ligands	Detoxification	(Gong et al., 2021a)
		Nano-sensor	
Modified cell membrane	Improved targeting	Cancer therapy	(Zhou et al., 2016; Jiang et al., 2019; Krishnamurthy et al., 2019; Park et al., 2021; Yu et al., 2022)
	Prolonged blood circulation	Drug delivery	
	Introduces or regulates functional membrane molecules		

CM-coated biomaterials have superior biocompatibility, decreased clearance by the reticuloendothelial system, prolonged circulation time, and improved colloidal stability and homotypic binding (Zhu et al., 2016; Liu et al., 2020, 2021; Sun et al., 2020; Zhao et al., 2020). The stabilizing effect of CM coating can be attributed to flexible and hydrophilic surface glycans on CMs. They provide steric hindrance to prevent aggregation (Rao et al., 2017). The bare core particle surface with high surface energy readily interacts with glycan-rich membranes to minimize the overall energy. Once stabilized with the glycan-rich CM coating, particles are rarely involved with further membrane interactions due to the stealth effect and electrostatic repulsions. This explains the likelihood of formation of unilamellar CM coating even in the presence of excess CMs (Luk et al., 2014). Additionally, CM coatings are immune-evasive and contain self-markers reducing immune clearance of synthetic cores (Hu et al., 2013).

**FIGURE 2:**
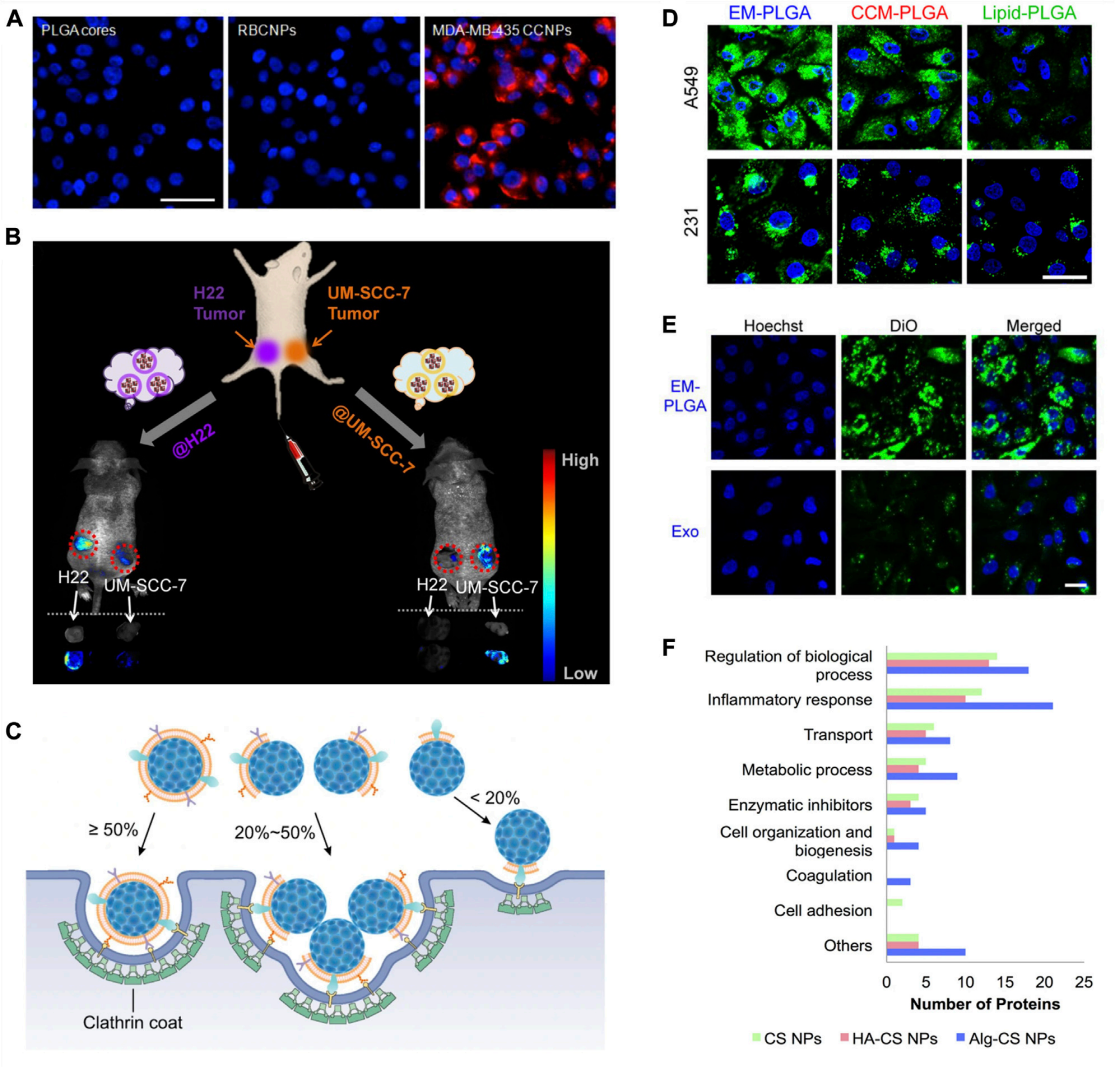
Biological membranes and biomolecules of surface modification. **(A)** Fluorescent imaging of MDA-MB-435 cells incubated with PLGA cores, RBCM-coated PLGA NPs (RBCNPs), or MDA-MB-435 tumor CM-coated NPs (CCNPs). NPs were labeled with DiD (red) and nuclei were stained with DAPI (blue). Scale bar, 50 μm. Reprinted with permission from Fang et al. (2014). Copyright 2014 American Chemical Society. **(B)** Schematic illustration of the *in vivo* study with H22 and UM-SCC-7 dual-tumor-bearing mouse model. Fluorescence images and *ex vivo* images of tumors at 12 h post injection with H22 and UM-SCC-7 tumor CM-coated magnetic NPs. Reprinted with permission from Zhu et al. (2016). Copyright 2016 American Chemical Society. **(C)** Illustration of different cell internalization strategies by NPs with different CM coating percentages by Liu et al. (2021) licensed under CC BY 4.0. **(D)** Confocal fluorescence images of cellular uptake of A549 cell-derived EM-coated (EM-PLGA), A549 cancer CM-coated (CCM-PLGA), and lipid-coated (lipid-PLGA) PLGA NPs by A549 and MDA-MB-231 (231). The PLGA cores were labeled with DiO (green), and the cell nuclei were stained with Hoechst (blue). Scale bar, 20 μm. **(E)** Confocal fluorescence images showing higher cellular uptake of EM-PLGA NPs compared to exosomes (Exo) by A549 cells. The membranes of EM-PLGA NPs and Exo were labeled with DiO (green). The cell nuclei were stained with Hochest (blue). Scale bar, 40 μm; Reprinted with permission from (C. Liu et al. (2019)). Copyright 2019 American Chemical Society. **(F)** Comparison of functions of protein corona of bare chitosan NPs (CS NPs), HA-coated CS NPs (HA-CS NPs), and alginate-coated CS NPs (Alg-CS NPs) by (Almalik et al., 2017) licensed under CC BY 4.0.

Homotypic binding is an attractive characteristic of CM coatings. It means preferential binding of CM-coated particles to their source cells *via* translocated cell receptors and adhesion molecules. Breast cancer cells, MDA-MB-435, were incubated with uncoated-, RBC membrane (RBCM)-coated, and cancer CM (CCM)-coated PLGA NPs (∼110 nm) (Fang et al., 2014). The cellular uptake of CCM-coated NPs was 20-fold and 40-fold higher than uncoated- and RBCM-coated NPs, respectively (Figure 2A). In another study, H22 CCM-coated magnetic NPs (∼102 nm) were intravenously administered to mice bearing both H22 and UM-SCC-7 tumors (Zhu et al., 2016). Accumulation of NPs was three times higher in the H22 tumor than that in UM-SCC-7 tumor (Figure 2B). When coating was switched from H22 CM to UM-SCC-7 CM, the trend was reversed.

In an investigation on the integrity of the CM coating, RBCM demonstrated the highest degree of coating over the platelet membrane, CCM, and macrophage CM because of the well-preserved membrane structure after a harsh isolation procedure (Liu et al., 2021). Despite the partial coating, CM-coated NPs (∼140 nm) still exhibited the homotypic binding and reduced phagocytosis. Based on TEM (transmission electron microscope) analysis and dissipative particle dynamics (DPD) simulations on cellular uptake, it was found that NPs with a high degree of coating (≥50%) enter the cells individually; NPs with a low degree of coating (20–50%) aggregate to hide uncoated surfaces in the interior while exposing coated surfaces to enter the cells, and NPs with a very low degree of coating (<20%) hardly enter the cells (Figure 2C). Functional proteins on CM coatings can be also used to perform the biochemical reaction in a spatially controlled manner. For example, in a RBCM-coated therapeutic protocell (∼5.4 µm), the authors utilized hemoglobin trapped on the CM for an enzymatic reaction that produced nitric oxide (Liu et al., 2020).

### Exosomes

Exosomes (40–150 nm) are lipid-bilayer-enclosed vesicles secreted by all types of cells (Lai et al., 2013; Samanta et al., 2018; Zhang et al., 2022). They originate from the endosomal compartments and exocytosis in response to cell activation or death. They are involved in cell–cell communications and carry nucleic acids, proteins, and metabolites. The exosome membrane (EM) is rich in membrane proteins for cellular targeting and trafficking that are derived from both endosome and plasma membranes (Zhu et al., 2018b). EM can be decorated to express noninherent molecules by genetically engineering parent cells, chemical conjugation, and lipid insertion (Tian et al., 2014; Kooijmans et al., 2016; Nakase et al., 2017; Fu et al., 2020; Han et al., 2020; M. Xu et al., 2020b).

Exosomes have been evaluated for their diagnostic and therapeutic potential in more than 200 clinical trials up to date (Huda et al., 2021). The exosomes present in a blood sample give an account of the parent cells, or they can be manipulated to deliver or interfere with therapies (Table 3). Nevertheless, the poor drug-loading capacity of exosomes of 30% against ∼90% of conventional NPs is clinically undesirable (Liu et al., 2019a). In addition, drug-loaded exosomes exhibit low Young’s modulus (∼10 MPa), which requires more energy to be internalized by cells (Liu et al., 2019a). To improve drug-loading capacity of exosomes, drug-loaded NPs can be encapsulated within the exosomes (Gangadaran and Ahn, 2020; Mehryab et al., 2020). In other words, the exosome is treated as a surface-modifying material of NPs. As all extracellular vesicle categories share similar structural properties (Samanta et al., 2018), the surface modification techniques of exosomes would be also applicable to all extracellular vesicles.

**TABLE 3:** Functions and application of some common exosomes.

Exosome source	Function	Application
Cancer cells (Yong et al., 2019; Mehryab et al., 2020)	Cancer metastasis	Cancer therapy
Macrophage (McDonald et al., 2014; Xu et al., 2020b)	Immunoregulatory functions	Cancer therapy
		Brain delivery
Mesenchymal stem cell (Kalimuthu et al., 2018; Nikfarjam et al., 2020)	Tissue regeneration	Regenerative medicine
	Immunomodulation	Autoimmune disease
		Cancer therapy
Dendritic cell O’Loughlin et al. (2012); Pitt et al., 2016; De Jong et al., 2019; Xu, Jia and Xu, 2019)	Immunomodulation	Immunotherapy
		Gene therapy
		Autoimmune diseases
Blood (Qi et al., 2016; Hornung, Dutta and Bitan, 2020)	Disease progression	Chemotherapy
		Diagnosis and monitoring treatment efficacy

Exosomes contain cell adhesion molecules from source cells and therefore promote homotypic binding (Zhu et al., 2018b). In a study on targeted delivery using lipid-coated, CM-coated, and EM-coated NPs, EM-coated NPs had the best performance despite the narrow protein profile compared to those of CM-coated NPs (Figure 2D). When EM-coated NPs were compared with the source exosome, rigid EM-coated NPs had 15.3-fold higher cellular uptake (Figure 2E). EM-coated NPs (∼130 nm) can be internalized in multiple pathways including caveolae-mediated endocytosis, clathrin-mediated endocytosis, and micropinocytosis (Xiong et al., 2019). Overall, EM-coated biomaterials could offer significant advantages in terms of long-distance intracellular communication, reduced immunogenicity, inherent therapeutic potential, long-term accumulation, and modifiable targeting and trafficking effect (O’Loughlin et al., 2012; Kalimuthu et al., 2018; Xiong et al., 2019; Han et al., 2020; Huda et al., 2021).

### Lipids and fatty acids

Lipids are the major component of biological membranes and are responsible for their bilayer structure and fluidity (Spector and Yorek, 1985). Hence, lipids have been widely used in constructing biological membrane mimics. Lipids are a variety of non-polar molecules including fatty acids, phospholipids, and sterols. Fatty acids are amphiphilic molecules with a long (un) saturated hydrocarbon chain and a carboxyl group at the end of the chain (Kumar, 2019). They are the building blocks to fabricate triglycerides, phospholipids, or cholesterol. Phospholipids consist of two hydrophobic fatty acid tails and a hydrophilic phosphate head. Over the last decades, researchers found and learned to synthesize different types of phospholipids for different functions. Cholesterol is the most popular sterol in lipid-based biomaterials (Nakhaei et al., 2021). It contains a 27-carbon molecule with a hydroxyl group to form hydrogen bonds with phospholipids. Table 4 includes some common lipids in biomaterials. Phase-transition temperature, hydrolytic stability, charge, and polymorphism are some important factors for lipid selection (Peetla, Vijayaraghavalu and Labhasetwar, 2013; Nakhaei et al., 2021). Lipids can be formed on biomaterials to render biocompatibility and biological functions, scaffold for further functionalization, and model plasma membrane (Cheung et al., 2018; Dunn, Mac and Wang, 2019; Luchini and Vitiello, 2021).

**TABLE 4:** Common lipid types, their characteristics, and uses.

Lipid type	Characteristic and use
DOPE	
(1,2-Dioleoyl-sn-glycero-3-phosphoethanolamine)	Zwitterionic, unsaturated phospholipid
	Common in cationic liposomes
(Mahato, Smith and Rolland, 1999; Prata et al., 2008)	Promotes cargo release by disrupting the endosome membrane
DSPE	
(1,2-Distearoyl-3-sn-glycerophosphoethanolamine)	Saturated analog of DOPE
Che et al. (2015)	DSPE-PEG is popular in lipid formulations
POPC	
(1-Palmitoyl-2-oleoyl-glycero-3-phosphocholine)	Monosaturated fatty acid composition mimics mammalian phospholipid composition
(Cheung et al., 2018; Hasani-Sadrabadi et al., 2018)	Used to model CM
DSPC	
(1,2-Distearoyl-sn-glycero-3-phosphocholine)	Zwitterionic, saturated phospholipid
Hou et al. (2021)	Widely used in forming lipid NPs for mRNA delivery as cylindrical geometry stabilizes the structure of lipid NP
DPPC	
(1,2-Dipalmitoyl-sn-glycero-3-phosphocholine)	Produces temperature-sensitive liposomes (phase transition temperature at ∼41°C)
(Kono and Takagishi, 2004; Li et al., 2020)	Lung surfactant, thus used in inhalation delivery
DOTAP	
(1,2-Dioleoyl-3-trimethylammonium-propane)	Cationic
Pedroso de Lima et al. (2001)	Popular in gene delivery
DOPS	
(1,2-Dioleoyl-sn-glycero-3-phospho-l-serine)	Anionic at pH 7.4
(Lentz, 2003; Gaul et al., 2015)	Incorporates in model platelet membranes
Ionizable lipids	Neutral at physiological pH and positive at low pH
(Hou et al., 2021)	Suitable for drug delivery applications
Cholesterol	Regulates cohesiveness, fluidity, and permeability of the lipid membrane
(Ruwizhi and Aderibigbe, 2020; Nakhaei et al., 2021)	Improves the thermal and plasma stability of liposomes

### Proteins and peptides

Proteins are biological macromolecules that are made up of different combinations of amino acids. They are essential in maintaining and regulating functions and structures of the body including cellular machinery, metabolic activities, cell signaling, molecular transportation, and immune system regulation (Balaji et al., 2015). The folded structure of a protein is vital to perform its task. Table 5 contains some common protein groups that have been widely studied for surface modification of biomaterials. Peptides that can be readily made by solid-phase synthesis were also used in surface modification (Li et al., 2011; Wang et al., 2014). Sometimes, a simple peptide chain can be used instead of a whole protein to attain the objective (Chesson et al., 2014; Kakwere et al., 2017). For example, the epitope SIINFEKL, an octamer peptide, can be used to represent the ovalbumin antigen because the epitope is the part of an antigen that is recognized by the immune system (Karandikar et al., 2019). Compared to proteins, surface modification with peptides is relatively simple since anchoring motifs can be incorporated during peptide synthesis. The requirement of specific binding orientations for peptides is less stringent (Pagel and Beck-Sickinger, 2017). Surface modification with proteins and peptides may assist in active targeting, boosting the immune system, spatial control of biochemical reactions, and improving plasma stability (Tebbe et al., 2015; Gibson et al., 2018; Rhodes and Green, 2018; Li, Liu and Zhao, 2022).

**TABLE 5:** Some common proteins and peptides of surface modification of biomaterials.

Protein or peptide category	Examples	Note
Plasma proteins	Albumin, fibrinogen, globulin, and transferrin	Improve hemocompatibility (Steinberg et al., 1989)
		Enhance plasma stability (Yeo et al., 2018)
		Reduce platelet adhesion (Neumann et al., 1979)
Extracellular matrix proteins	Collagen, elastin, fibronectin, vitronectin, and laminin	Improved cell adhesion (Chen, Kawazoe and Tateishi, 2008)
		Increased wettability (Balaji et al., 2015)
Antibodies	Anti-CD3 and anti-CD28	T-cell activation (Neal et al., 2017)
	Anti-EGFR, anti-HER2, and anti-EpCAM	Target tumor cells (Gibson et al., 2018; Oh et al., 2018; Ai et al., 2021)
Antigen	Ovalbumin	Model antigen (Wang and Mooney, 2018)
Peptides	RGD motif containing peptides	Cell adhesion (Pagel et al., 2016; Buxadera-Palomero et al., 2017)
		Tumor accumulation (Rios De La Rosa et al., 2020)
	NGR motif containing peptides	Tumor tropism (Graziadio et al., 2016)
	Repeated sequence of PSA	Stealth effect (Schlapschy et al., 2013)
	E14LKK/H14LKK	Antimicrobial activities (Balaji et al., 2015)
	SIINFEKL	Model epitope (Karandikar et al., 2019; Kramer et al., 2019)

### Polysaccharides

Surface modification with polysaccharides provides a hydrophilic interface on to hydrophobic biomaterials (Park et al., 2011). These natural polymers are long chains of mono/disaccharide units linked with glycosidic bonds. Chitosan, hyaluronic acid (HA), dextran, and alginate are the most studied polysaccharides for biomaterial design. Being abundant in the extracellular matrix (ECM), polysaccharides interact with cells, organs, and tissues in unique ways (Table 6) (Berry et al., 2003; Bravo-Osuna et al., 2007; Lima, Sher and Mano, 2012; Singh and Peppas, 2014; Monette et al., 2016; Pistone et al., 2017; Ramos Avilez et al., 2017; Liu et al., 2018; Duan, Chan and Lin, 2019; Chung et al., 2021; Mukwaya et al., 2021; Zeng et al., 2021; Della Sala et al., 2022). Furthermore, polysaccharide coatings can affect the colloidal stability and cargo release from the core particle (Chronopoulou et al., 2013). A study compared the composition of protein corona of bare, HA-coated, or alginate-coated chitosan NPs (Figure 2F) (Almalik et al., 2017). HA-coated NPs demonstrated the lowest total protein binding. Moreover, protein composition was dependent on the coating. HA coating was the least immunogenic with less inflammatory proteins adsorbed, while bare and alginate-coated NPs selectively adsorbed proinflammatory proteins.

**TABLE 6:** Features and cellular modulation characteristics of common polysaccharides.

Polysaccharide	Cellular modulation characteristic	Feature
Chitosan	Antimicrobial activity	Cationic
		Bio-adhesive
	Stimulates M1 macrophages	pH-responsive
	Expands antitumor T-cell population	High biodegradability
Hyaluronic acid (HA)	Inherent tumor tropism by HA-binding receptors	Major component of ECM
	Stimulates dendritic cells	Anionic
		Non-toxicity
Dextran	Promotes dendritic cell maturation	High biodegradability
	Dextran is a biocompatible alternative to PEGylation	Non-toxicity
Alginate	Highly interactive with macrophages	Bio-adhesive
		Easy functionalization

## Techniques of surface modification

Mild and non-denaturing processes, namely, biorthogonal chemistry, coextrusion, sonication, and adsorption, are the most commonly reported methods in surface modification of micron- and nano-scale biomaterials using natural materials (Zhang et al., 2019; Zeng and Pu, 2020). This section will cover different surface modification techniques available not only for solid biomaterials but also for emulsion droplets. Usually, cell membranes, exosomes, and lipid vesicles share the same set of techniques as coextrusion, sonication, and electroporation owing to structural similarities (Liu et al., 2019a; Sancho-Albero et al., 2019b). Macromolecules such as proteins and polysaccharides can be directly (without preparation of vesicles) used in coatings. Their shared coating techniques include adsorption and chemical conjugation.

### Coextrusion

Up to date, coextrusion is the main method of coating NPs using CMs (Li et al., 2018a; Yang et al., 2021b; Zhao et al., 2021). A negative surface charge, smaller size, and high CM concentration may lead to a complete CM coating in the coextrusion process (Luk et al., 2014; Liu et al., 2021). Furthermore, EM- and lipid-coated NPs could be prepared by coextrusion (Messerschmidt et al., 2009; Jo et al., 2014; Zeng et al., 2018; Van Deun et al., 2020). The core particles and preformed CM, EM, or lipid vesicles will be extruded multiple times through a series of porous polymer films. This process uses shear force to deform/rupture vesicles and allow them to reassemble around the cores in an energetically favorable process. The ratio between vesicles and NPs can be manipulated to control uniformity, thickness, and stability of the coating (Cheng et al., 2018). In a systemic study of determining feasibility of coextrusion to coat NPs with EM, 87% of the total number of membrane protein types, 70% of the total number of protein types, and the orientation of EM could be preserved during extrusion (Van Deun et al., 2020). However, Fuhrmann et al. reported that extrusion changes the charge, constitution, and targeting activities of the EM (Fuhrmann et al., 2015).

Coextrusion has advantages such as high degree of control and reproducibility. However, coextrusion is unfavorable in scaling-up. Clogging is the major disadvantage of coextrusion. Clogging depends on the concentration and composition of the core-vesicle mixture, flow rate, temperature, and pressure (Cullis, Hope and Bally, 1991). High-concentration solutions and oppositely charged cores and vesicles may aggregate and impede/clog the extrusion process. Temperature should be high enough to attain the “fluid state” of lipids, and pressure and flow rate should be optimized depending on solution composition and geometry of equipment. Under extruder membranes, the “tortuous path”-type membrane (polyether sulfone membrane) which consists of a fiber matrix can easily be clogged up compared to the “nucleation track”-type membrane (polycarbonate membrane) which is a thin polymer sheet with straight holes of exact diameter (Ong et al., 2016). To coat metal–organic framework NPs with cancer cell-derived EMs, Cheng et al. combined sonication (1 min) and coextrusion (11 times) (Cheng et al., 2018). Pre-treatment with sonication reduced the risk of clogging during coextrusion.

### Sonication

**FIGURE 3:**
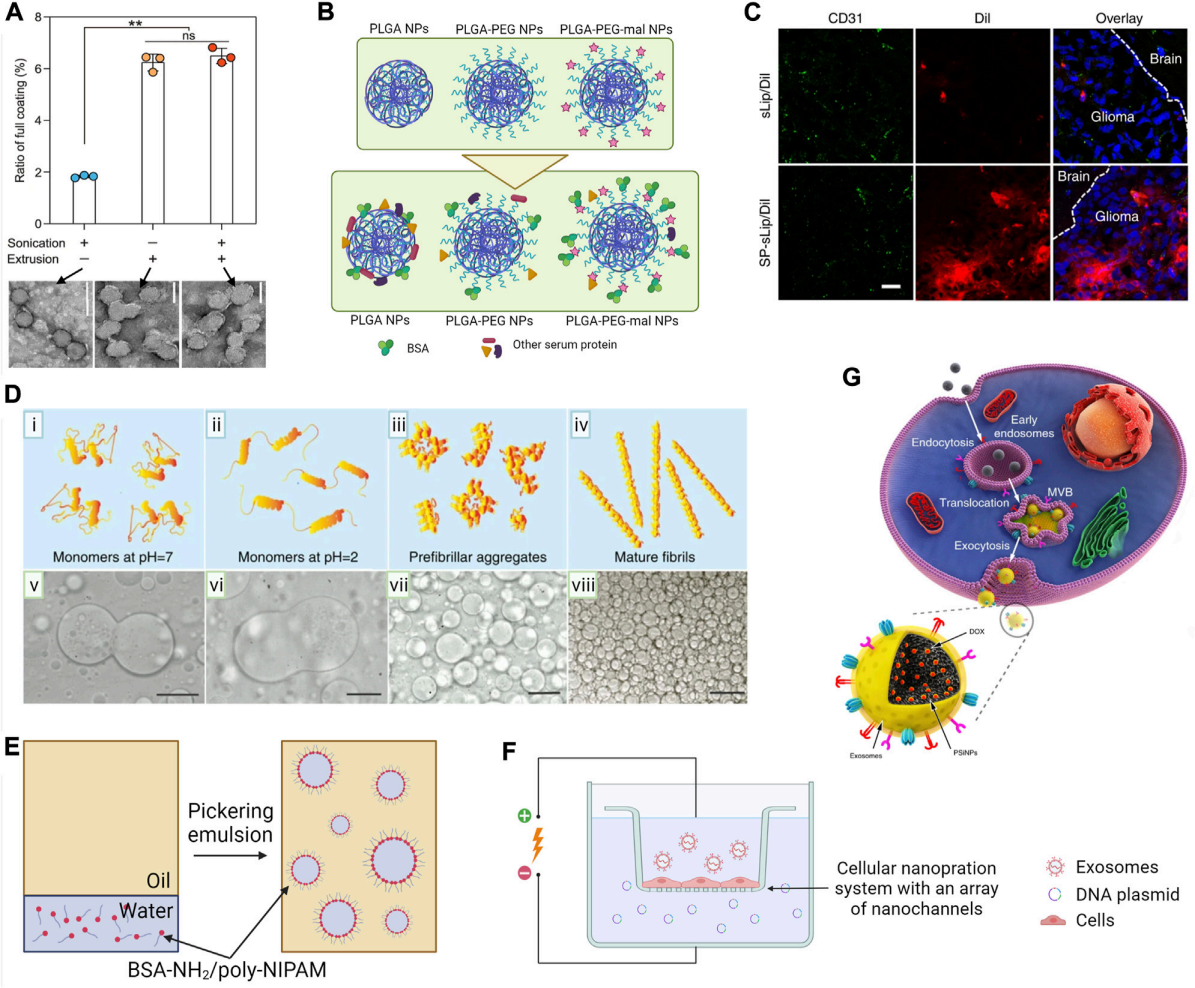
Surface modification techniques of micro- and nano-scale biomaterials. **(A)** Ratio of full CM coating accomplished with either sonication, extrusion, or combined sonication-extrusion and TEM images of each sample. Scale bars, 100 nm; by Liu et al. (2021) licensed under CC BY 4.0. **(B)** Schematic illustration of the *in situ* coating protein on PLGA NPs *in vivo*. Bare, PEGylated (PLGA-PEG), and maleimide-conjugated (PLGA-PEG-mal) PLGA NPs (at top) will acquire a protein corona during *in vivo* circulation (at bottom). Protein corona of PLGA-PEG-mal NPs was enriched with albumin that was covalently conjugated to maleimide motifs (Li et al., 2018d). **(C)** Confocal microscope images of brain slices at 4 h after liposome administration via the tail vein of nude mice bearing intracranial glioma. Brain slices were stained with the anti-CD31 antibody (green) and biodistribution of liposomes (red) in normal brain tissues (labeled with the brain) and glioma region (labeled with glioma) by Zhang et al. (2019) licensed under CC BY 4.0. **(D)** Stability of dextran-in-PEG emulsions by adding lysozyme in different aggregation stages as (i–iv) graphical representation of lysozyme aggregation state and (v–viii) corresponding optical micrographs showing stabilization behavior. Scale bars, 50 µm by Song et al. (2016) licensed under CC BY 4.0. **(E)** Procedure for preparation of water-in-oil proteinosomes using BSA-NH2/PNIPAAm nanoconjugates. An aqueous suspension of amphiphilic BSA-NH2/poly-NIPAM nanoconjugates was emulsified in a continuous oil phase. Nanoconjugates spontaneously self-assembled around water-in-oil emulsions to produce stabilized protein-based spherical microcompartments (Huang et al., 2013). **(F)** Schematic representation of nanoporation-based exosome generation. A monolayer of parent cells will culture above an array of nano-channels (approximately 500 nm in diameter). Plasmid DNA in the buffer enters the attached cells through nanochannels under transient electrical pulses. Attached cells subsequently release large quantities of exosomes containing transcribed mRNA (Yang et al., 2020). **(G)** Schematic illustration of the preparation of EM-coated NPs (PSiNPs). PSiNPs are endocytosed into cancer cells after incubation, and then localized in multivesicular bodies (MVBs) and autophagosomes. After MVBs or amphisomes fuse with the cell membrane, EM-coated PSiNPs are exocytosed by Yong et al. (2019) licensed under CC BY 4.0.

Sonication is a simple method with a higher throughput and less loss of material compared to coextrusion. In sonication-mediated coating of CM, EM and lipids, acoustic waves, and cavitation bubbles lead to a spontaneous fusion of the core material and preformed vesicles (Liu et al., 2019a; Chai et al., 2019; Lv et al., 2021; Wang et al., 2021). Simultaneously, sonication pulses temporarily decrease membrane microviscosity and enhance membrane permeability, but do not disturb proteins or lipids of the vesicle (Kim et al., 2016; Xiong et al., 2019). However, extrusion is more efficient than sonication in the formation of a full CM coating (Figure 3A) (Liu et al., 2021). Yang et al. argued that optimization of the sonication procedure may surpass coextrusion as the low yield of coextrusion is commercially undesired (Yang et al., 2021b). They investigated the effect of amplitude and duration of sonication, density of particles, and temperature on the efficiency of coating NPs. Some important findings of the study are 1) sonication at lower temperatures will result in stable coatings, 2) extended durations and higher amplitudes lead to an unstable coating, and 3) low NP density leads to poor coating efficiency and stability.

Fatty acid coatings can promote colloidal stability and enhance the surface crystalline quality of magnetic iron oxide NPs (Bixner et al., 2015; Salvador et al., 2022). Sonication is used to coat magnetic NPs with fatty acids (Argüelles-Pesqueira et al., 2018; Gupta et al., 2019). In a study assessing five types of fatty acid coatings, the coating was found to reduce the magnetic properties of the NPs depending on the hydrophobicity of the hydrocarbon chain (Ong, Suppiah and Muhd Julkapli, 2020). An increase in sonication time reduced polydispersity and hydrodynamic radius of NPs due to homogenous coating. However, over-sonication may cause aggregation. The authors concluded that oleic acid is the best fatty acid coating agent to prevent agglomeration compared to capric acid, stearic acid, palmitic acid, and myristic acid. Compared to other saturated fatty acids, oleic acid is more hydrophilic due to its double bond. Hence, oleic acid-coated NPs formed stable dispersions with thin fatty-acid coating, smallest hydrodynamic size, and lowest polydispersity index.

Sonication can be used to replace the synthetic surfactant coating of NPs with a protein layer. Tebbe et al. replaced cetyltrimethylammonium bromide (CTAB) surfactant coating with BSA coating by sonication (Tebbe et al., 2015). The authors used three strategies for concurrent destabilization of CTAB and coating with BSA: 1) maintaining a high BSA-to-CTAB ratio to shift equilibrium toward BSA-coated particles, 2) removal of released CTAB by centrifugation in the middle of coating, and 3) vigorous mixing *via* sonication. Even though rarely reported, sonication might be useful in coating colloidal biomaterials with proteins.

### Adsorption

Adsorption would be the simplest method of surface modification of biomaterials. It has been widely reported in surface modification with lipids (Khan et al., 2017), polysaccharides (Yugay et al., 2020), and proteins (Yeo et al., 2017; Yeo et al., 2018). Adsorption of lipid layers on solid supports involves three major steps, that is, 1) adsorption of lipid vesicles, 2) deformation and subsequent rupturing of vesicles spreading into bilayer patches, and 3) fusion of bilayer patches to produce a continuous membrane (Mornet et al., 2005). For successful lipid layer deposition, lipid vesicles should have a large affinity toward the surface through long-range electrostatic interactions, along with short-range chemical and van der Waals interactions. Lipids should be capable of lateral mobility to rearrange along the support surface to form a continuous membrane, and hence, strong hydrogen bonds are undesirable (Fischlechner et al., 2008). This precise balance between interactions not only depends on the support and the lipids, but also on buffer composition, ionic strength, pH, and temperature. Generally, temperature needs to be maintained above the phase-transition temperature of lipids (Khan et al., 2017). Even though adsorption is not generally considered in surface modification with CM and EM, the process would be similar to that of lipids (He and Park, 2016; Zhang, Ma and Wei, 2021).

Physical adsorption of polysaccharides onto micron- and nano-sized biomaterials occurs by electrostatic and hydrogen bonds (Park et al., 2011; Atoufi et al., 2019). A one-pot synthesis method was reported for polysaccharide-coated gold, silver, and copper NPs (Huang and Yang, 2004; Yugay et al., 2020). Here, polysaccharide plays a dual role of the reducing agent and stabilization agent. At high pH, cationic metal ions will be reduced to their elemental state through the oxidation of the hydroxyl end group of polysaccharide to the aldehyde group (Stiufiuc et al., 2013). Simultaneously, the polysaccharide stabilizes the produced colloidal particles, forming a non-covalent coat around them. The concentration and length of polysaccharide influence the morphology and size distribution of the NPs (Huang and Yang, 2004; Luo et al., 2005).

The natural occurrence of the physical adsorption of a protein coating is called “protein corona.” When a biomaterial is planted in a biological environment, depending on the size, material, and surface charge of the biomaterial, resident proteins will immediately form a “protein corona” around the material (Rampado et al., 2020). This phenomenon involves electrostatic, hydrophobic, or hydrogen bond interactions (Yu and Chen, 2015). Although protein corona can be a biological barrier to colloidal stability and immunogenicity of biomaterials, it can also be a coating to improve biocompatibility. Yeo et al. non-covalently modified gold nanorods (46.5 ± 1.2 nm by 19.0 ± 0.7 nm) with serum proteins by simple incubation (Yeo et al., 2017; Yeo et al., 2018). They observed spontaneous assembly of proteins around nanorods, later improving the colloidal stability of the particles in *in vivo* studies. To improve protein adsorption kinetics and strength, the biomaterial surface can be decorated with protein-binding anchors (Mertz et al., 2011, 2012). Shi et al. used tannic acid-doped calcium carbonate particles (∼5 µm) to adsorb proteins onto their surface (Shi et al., 2019). Tannic acid penetrates the hydrophobic pockets of the protein surface, forming hydrophobic–hydrophobic interactions. Hydrogen bonds can be formed between phenolic hydroxyl groups of tannic acid and polar groups of proteins. Electrostatic interactions are formed between hydroxyl groups of tannic acid and amino groups of proteins in a pH-dependent manner. This multimode binding allowed proteins to be absorbed regardless of the molecular weight, isoelectric points, amino acid sequence, solubility, and functional domains. The authors used 10 different proteins with sizes ranging between 12–660 kDa and isoelectric points ranging between 4.6–10.8 to demonstrate the universality of the method. The selected molecular weight of the protein determined the protein layer thickness. In another work, small bromo*iso*butyramide molecules were used to modify silica microparticles (MPs) through hydrogen bonds between amide groups and halogen bonds of bromines (Mertz et al., 2011). The diversity of the amino acid sequence allows proteins to interact with different molecules using different interactions.

Chemisorption is an adsorption process that involves sharing of electrons between the surface of the adsorbent and adsorbate, forming covalent or ionic bonds. This process is commonly used in protein-based surface modifications. A chemically adsorbed protein layer is more stable and irreplaceable compared to physical adsorption (Nakata et al., 1996). If necessary, proteins can be chemically or genetically engineered to bind to intrinsic motifs of the material surface (Pagel and Beck-Sickinger, 2017). Thiol-mediated surface modification of gold (Nakata et al., 1996), silanes, phosphonate- or amine-mediated surface modification of metal oxides (Pujari et al., 2014), and neutrophilic amine-mediated surface modification of noble metals (Reymond-Laruinaz et al., 2016) are some examples of protein chemisorption. Both physical and chemical adsorption of proteins are dependent on the concentration of the protein and binding motifs, temperature, pH, solvent, and incubation time (Nakata et al., 1996).

### Chemical conjugation

Covalent binding through click chemistry is widely used in functionalizing biomaterial surface with proteins and peptides (Zhou et al., 2017; Marques et al., 2020). The rapid Michael addition reaction between the thiol group of cysteine and the maleimide group on the biomaterial is the most common click chemistry reaction in decorating the biomaterial surface with peptides and proteins (Wang et al., 2014; Ravasco et al., 2019). Conjugation efficiency of peptides can be significantly high even compared to small proteins (Martínez-Jothar et al., 2018). Functionalizing lipids with peptides prior to liposome formation can increase conjugation efficiency by >10% compared to post-functionalization (Feldborg, Jølck and Andresen, 2012). An interesting *in situ* protein coating on PLGA NPs using click chemistry was reported by Li et al. (2018d). The authors decorated NPs with PEG–maleimide moieties that can selectively react with the cystine-34 residue of endogenous albumin through the Michael addition reaction (Figure 3B). Maleimide functionalization tripled the albumin content of protein corona. These NPs demonstrated a comparable circulation half-life as PEG-functionalized NPs. Importantly, accelerated blood clearance of usual PEGylated NPs could be avoided by outer albumin corona. Another study used non-neurotoxic β-amyloid_25-35_ peptide-modified liposomes for *in situ* adsorption of a plasma protein coating that can cross the blood–brain barrier (BBB) (Zhang et al., 2019). Cystine-terminated peptides attached to maleimide lipids, prior to liposome preparation through the Michael addition reaction. This peptide interacted with lipid-binding domain of apolipoproteins in plasma, exposing receptor-binding domains to the external environment. Through apolipoprotein-specific receptors are present on BBB and glioblastoma cells, peptide-functionalized liposomes exhibited 14.5- and 44-fold higher accumulation in the brain and glioma, respectively (Figure 3C). *In situ* protein coatings allow self-protein adsorption, reduce immunogenicity, and avoid complex surface modification operations.

Hydroxyl-rich polysaccharide coating can be used as a scaffold to add various functionalities to the material surface (Unterweger et al., 2014; Wang et al., 2016). Stolyar et al. covalently attached biotin and streptavidin with amino and hydroxylgroups of chitosan-coated magnetite NPs (Stolyar et al., 2021). These immobilized molecules can provide strong linkages and selectivity for further surface functionalization. Chemical and structural diversity of polysaccharides (chain length, monosaccharide sequence, stereochemistry, etc.) provide a wide range of tools to develop multifunctional coating for biomaterials. A number of polysaccharide derivatives that may covalently bind with amines, thiols, carbonyls, esters, etc. on biomaterial surfaces have been developed over the past years. These derivatives can change the hydrophilicity and cellular modulation characteristics of native polysaccharides (Chen and Huang, 2018).

In an interesting study, serum-derived exosomes were used to modify the surface of microspheres, not to target or to remove immunogenicity but to increase surface roughness (You et al., 2020). High surface roughness of the MPs may facilitate phagocytosis due to the increased contact area and adhesive forces (Champion, Walker and Mitragotri, 2008). Exosomes were chemically conjugated to polydopamine-coated PLGA microspheres through the Michael addition reaction. The authors showed about 9-fold and 2-fold higher cellular uptake of rough PLGA microspheres by DC2.4 and RAW264.7 cells, respectively. However, in the authors’ previous study of modifying surface roughness using CCM vesicles, cellular uptake by RAW264.7 was improved by 17.5-fold compared to the bare microsphere (Jung et al., 2019). This indicates the importance of selecting the cell type for coating. Immunogenic CCM is more efficient in triggering phagocytosis than non-specific exosomes.

### Pickering emulsion

Emulsion droplets could be wrapped with CMs, lipids, proteins, and polysaccharides by Pickering emulsion (Crowe and Keating, 2018; Douliez, Perro and Béven, 2019; Liu et al., 2020; Sakuta et al., 2020; Mu et al., 2021). In Pickering emulsion, vesicles or colloids of the coating material will adsorb on the interface of emulsions to lower free energy. It prevents coalescence, improving emulsion stability. Song et al. showed the importance of the size of colloids in Pickering emulsion in their work on stabilizing dextran-in-PEG droplets (10–150 µm) using lysozyme (Song et al., 2016). While small lysozyme monomers were partitioned into dextran-rich droplets, large lysozyme nanofibrils settled on the dextran-PEG interface (Figure 3D). As interfacial adsorption energy is proportional to the square of the radius of colloidal particles, large colloids tend to adsorb onto the emulsion droplet, whereas smaller colloids either remain in the continuous phase or partition into the droplets (Chao and Shum, 2020).

Uniformity of the coatings depends on whether the vesicles/colloids are stable or aggregative in the continuous phase (Pir Cakmak, Grigas and Keating, 2019). The vesicle/colloid layer is usually either sub-mono- or monolayer because the increase in vesicle/colloid concentration usually increases the number of emulsion droplets, reducing the diameter of droplets and increasing interfacial area, rather than forming multilayers (Huang, et al., 2013; Dewey et al., 2014; Cacace et al., 2015; Song et al., 2016). In a study of coating positive, negative, or neutral coacervates with lipid vesicles with different charges, sizes, and membrane fluidities, Lin et al. observed that lipid vesicles inherently tended to permeate into coacervates unless they are larger or rigid (Lin et al., 2020). Interestingly, charge interactions had little effect in determining whether the lipid vesicles stay on the coacervate surface or partition into the coacervate. For example, neutral or cationic lipid vesicles were compartmentalized by the cationic coacervate, whereas anionic lipid vesicles remained on the coacervate surface.

Generally, surface coatings produced by Pickering emulsion would not entirely inhibit the material exchange with the surrounding environment (Liu et al., 2020). In fact, membranes made by Pickering emulsion are popular in wrapping artificial cells as they display semipermeable properties as cell membranes (Crowe and Keating, 2018; Liu et al., 2020; Sakuta et al., 2020). Keating and coworkers used negative PEGylated lipid vesicles to coat dextran-in-PEG droplets (∼7 µm) (Dewey et al., 2014; Cacace et al., 2015). Despite the coating, nucleic acids, minerals, and enzymes were enriched within the droplet. However, particles larger than ∼130 nm diameter were excluded, demonstrating semi-permeability of the wrapping. When the ionic strength of the system was increased by NaCl, a higher vesicle packing density was observed due to shielding of electrostatic repulsions. The ability to control the packing density by salt might be useful for controlling the permeability of the coating.

In a similar study, a research team invented “proteinosome,” a water-in-oil emulsion droplet (20–50 µm) coated *via* interfacial assembly followed by crosslinking of BSA-NH_2_/poly (N-isopropylacrylamide) (poly-NIPAM) nano-conjugates (Figure 3E) (Huang, et al., 2013). Proteinosomes were semipermeable for polysaccharides of molecular weight less than 40 kDa and exhibited a temperature-sensitive permeability to hydrophilic compounds. Above 32°C, poly-NIPAM becomes hydrophobic, reducing membrane hydrophilicity and hence the permeability of hydrophilic compounds. In a later study, the authors modulated membrane porosity and consequently controlled the rate of reducing agent-triggered release of encapsulated DNA by differential crosslinking of the protein membrane using cleavable and non-cleavable agents (X. Huang et al., 2014b).

Polysaccharides have also been assessed to coat all aqueous emulsions. In a study on stabilizing all aqueous dextran-poly (ethylene oxide) emulsions using a linear homopolymer film, the authors evaluated the stabilizing effect of more than 10 different polysaccharides (Tea, Nicolai and Renou, 2019). Polysaccharides with both charged and hydrophobic groups (e.g., chitosan, diethyl aminoethyl dextran, and propylene glycol alginate) had strong emulsion stabilizing effects, while neutral hydrophobic polysaccharides and polyelectrolytes failed to prevent coalescence. The role of the charge in the stabilizer must be to provide the repulsive force against coalescence to maintain stability. However, the requirement of hydrophobicity is not clear, and the possible reason must be maintaining the integrity of the polysaccharide film.

### Electroporation

Electroporation breaks down the dielectric layer over CM or EM, and forms transient pores permitting internalization of NPs (Gehl, 2003). When electroporation was used to load NPs into exosomes, it led to a low encapsulation yield and a significant damage to the exosomes’ morphology with the formation of large aggregates through electrofusion (Sancho-Albero et al., 2019b). Hood et al. minimized exosome aggregation of electroporation by adding trehalose into the pulse medium and loaded magnetic NPs (5 nm) into melanoma exosomes (Hood, Scott and Wickline, 2014; Hu, Wickline and Hood, 2015). Trehalose is a sugar additive commonly used during liposome freeze-drying as a membrane stabilizer. Therefore, addition of trehalose not only prevented aggregation during electroporation, but also improved the stability of the EM. Yang et al. developed the nanoporation method, a modified electroporation to stimulate cells to produce and release exosomes containing exogenous RNA using controlled focal electrical pulses (Figure 3F) (Yang et al., 2020). They generated up to 50-fold more exosomes with more than 1000-fold increase in mRNA loading depending on the voltage. Nanoporation would be an alternative to overcome aggregation associated with traditional exosome electroporation.

### Passive loading

The passive loading method is more suitable for nano-size biomaterials and common in exosome-based surface modifications (Luan et al., 2017). However, encapsulation efficiency of the passive loading method can be as low as 20%. Even with the assistance of surfactants such as saponin and triton, the efficiency can only increase by a few percent (Sancho-Albero et al., 2019b; Fu et al., 2020). Generally, surfactants are useful in increasing the loading efficiency of small molecular weight drugs into exosomes (Fuhrmann et al., 2015; Haney et al., 2015). Thus, the size of the NPs appears to be a major limitation in the passive loading method.

Another form of passive loading is incubation or labeling parent cells with cargo particles and allowing them to be encapsulated when the parent cells produce exosomes through invagination (Figure 3G) (Neubert and Glumm, 2016; Altanerova et al., 2017; Luan et al., 2017; Yong et al., 2019). Usually, cargo material itself could stimulate the production of exosomes through autophagy. In a study of producing silica NP-loaded exosomes, when NPs were incubated with Atg7 (a crucial autophagy gene)-deficient fibroblasts, exocytosed NP% dropped, showing the autophagy dependency (Yong et al., 2019). When using the passive loading method to load drugs into exosomes, drug/exosome proportion, drug hydrophobicity, and exosome origin influence the loading efficiency (Fuhrmann et al., 2015; Mehryab et al., 2020). One may suggest that the same factors affect the passive loading of NPs into exosomes.

### Freeze-thawing

Freeze-thawing has been evaluated to encapsulate NPs within exosomes, but will also be applicable to encapsulate NPs within CM and lipid vesicles. However, repeated freezing might damage the EM integrity and show exosome aggregation (Haney et al., 2015). In a study of two different freeze-thawing cycles such as 1) −80°C for 30 min and defrosting and 2) 42°C for 30 s and 4°C for 2 min, encapsulation efficiencies were reported as ∼18% and ∼9%, respectively (Sancho-Albero et al., 2019b). Hence, further improvements are needed in freeze-thaw cycle-assisted coating.

### Coprecipitation

In coprecipitation, the polysaccharide will be added to the reactant mixture of core particles. Polysaccharides coat around precipitating NPs by chemisorption (Anbarasu et al., 2015; Fu et al., 2017). They may control the growth of the core particle by confining the space available for crystal growth without affecting the crystalline structure (Castelló et al., 2015; Hauser et al., 2015). Hence, the concentration and timing of polysaccharide addition affect the particle size and polydispersity. High polysaccharide concentration is undesirable as small size and thick polysaccharide coating negatively affect the magnetic properties (Dutz et al., 2007; Unterweger et al., 2014; Shaterabadi, Nabiyouni and Soleymani, 2017). These trends are independent of the polysaccharide type. The coprecipitation has the advantage of easy scale-up.

### Emulsion evaporation

Emulsion evaporation is another method to encapsulate particles in polysaccharide and protein coatings (Martínez Gómez et al., 2008; Tang et al., 2016). First, an emulsion of particles and polysaccharides will be formed through vigorous stirring/sonication and then, evaporation of the oil phase under atmospheric pressure or lower pressure precipitate polysaccharide-coated particles. Double emulsion evaporation is a similar process but with two emulsification processes (Chen et al., 2017). Lemarchand and coworkers used single emulsion evaporation to fabricate core-shell NPs using a hydrophobic poly (e-caprolactone) (PCL)-dextran hybrid (Lemarchand et al., 2006). An increase in dextran% in the hybrid reduced the average diameter of the particles. Use of dextran with two different molecular weights (5 k and 40 k Da) produced two different packing conformations. Dextran-5k led to a compact and dense coating, while dextran-40 k produced a large and flexible loop in the shell. When incubated with plasma, composition of the protein corona of each particle was distinct. Loosely packed dextran-40k mainly adsorbed apolipoproteins, whereas the dextran-5k-coated and uncoated particles favored immunoglobulins.

### Coaxial electrospray

**FIGURE 4:**
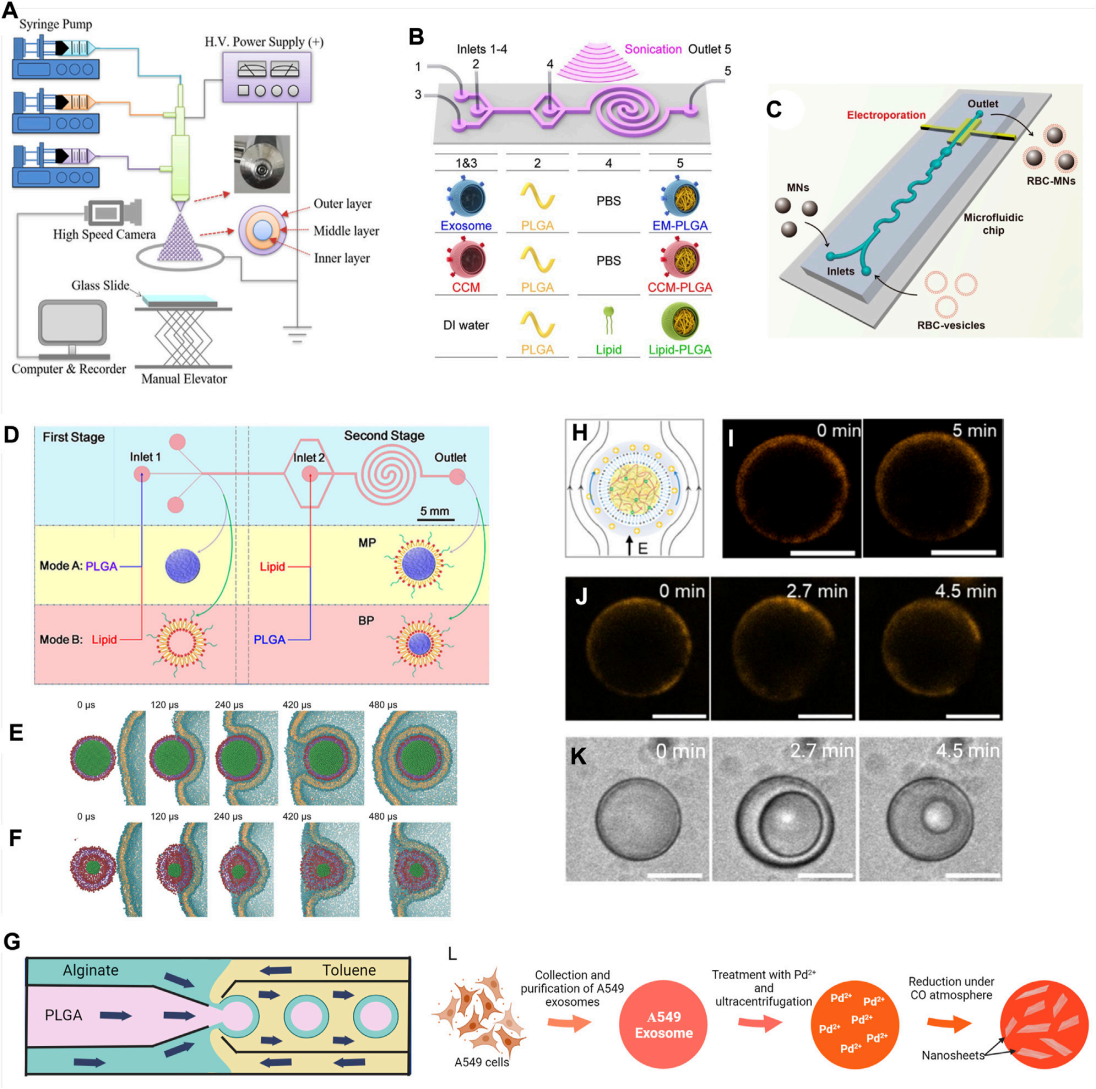
Surface modification techniques of micro- and nano-scale biomaterials. **(A)** Schematic diagram of a tri-axial electrospray system to produce multilayer core-shell particles. Reprinted with permission from Yao et al. (2020). Copyright 2020 American Chemical Society. **(B)** Schematic representation of the microfluidics sonication method for synthesis of the A549 exosome membrane (EM)-, cancer cell membrane (CCM)-, and lipid-coated PLGA NPs through the combined effects of sonication and microfluidics. The microfluidics device consists of one straight channel and one spiral channel connected with four inlets (inlets 1–4) and one outlet (outlet 5). The device is immersed in an ultrasonic bath, and the generated EM-PLGA NPs, CCM-PLGA NPs, and lipid-PLGA NPs are collected from outlet 5. Reprinted with permission from (C Liu et al., 2019). Copyright 2019 American Chemical Society. **(C)** Microfluidics electroporation-facilitated synthesis of RBCM-coated magnetic NPs (RBC-MNs). Reprinted with permission from Rao et al. (2017). Copyright 2017 American Chemical Society. **(D)** Schematic representation of the two-stage microfluidics chip to produce monolayer (MP) and bilayer (BP) lipid shell for PLGA NPs. Reprinted with permission from Zhang et al. (2015). Copyright 2015 American Chemical Society. Use of molecular dynamics (MD) simulation to demonstrate the influence of rigidity in cellular uptake of **(E)** MP and **(F)** BP. Reprinted with permission from Sun et al. (2015). Copyright 2014 WILEY-VCH Verlag GmbH & Co. KGaA, Weinheim. **(G)** Schematic diagram showing fabrication of PLGA–alginate core–shell microspheres using a multi-capillary microfluidics chip (Wu et al., 2013). **(H)** Illustration and **(I)** confocal fluorescence images of fatty acid-coated coacervate droplets excited at 10 V/cm showing membrane (BODIPY 558/568 C12—orange) slipped at the direction of green arrows. **(J)** BODIPY 558/568 C12 (orange) labeled fatty acid-coated coacervate droplets at 20 V/cm and **(K)** simultaneous repetitive cycles of vacuolization at that voltage. Scale bar, 10 μm. Reprinted with permission from Jing et al. (2019). Copyright 2019 American Chemical Society. **(L)** Illustration of preparation of Pd-nanosheets encapsulated exosomes from A549 cells (Sancho-Albero et al., 2019a).

The coaxial electrospray method prepares core-shell particles regardless of hydrophilicity or hydrophobicity of adjacent layers (Figure 4A) (Choi et al., 2010; Cao et al., 2014; Kim, Patel and Patel, 2021). Volatile solutions of core and shell materials will be sprayed onto a collector through a coaxial capillary needle with a high voltage applied between the needle and the collector. This forms a multi-layered Taylor cone and consequently, multilayered liquid droplets. Upon the flight to the collector, fast evaporation of solvent occurs, hardening particles. Particles of this technique usually have a narrow size distribution and minimal particle aggregation, owing to electrostatic repulsions between charged liquid droplets. In a recent work, a water bath was used to collect particles instead of the traditional hard aluminum foil/glass collector to preserve the morphology of particles (Tang et al., 2022). Chen and coworkers argued the importance of using same or miscible solvents for each layer in this method (Chen et al., 2018). They demonstrated that solvent diffusion between layers during the flight to the collector, even for a short time, is important to form a stable core-shell structure through partial insertion of shell material into the core layer. The other crucial parameters are 1) voltage which controls the formation of the Taylor cone, 2) flow rate which controls the particle size and morphology, and 3) distance of flight which is important to allow sufficient hardening of particles (Ghayempour and Mortazavi, 2013; Yao et al., 2020; Tang et al., 2022). Core-shell particles exhibit a significantly different packing density between layers because rapid solvent evaporation leads to a loosely packed shell and a dense shrank core.

### Electrostatic deposition

Joye et al. coated positively charged gliadin-based protein NPs with two different anionic polysaccharides at low pH by electrostatic deposition (Joye, Nelis and McClements, 2015). The authors discussed the significance of optimizing the ratio of polysaccharides/NPs in coating. An insufficient amount of polysaccharide may result in bridging flocculation, while excess amount of polysaccharide will result in depletion flocculation. The thickness of coating depends on the conformation of the adsorbed polysaccharides (Pistone et al., 2017). Polysaccharides with high charge densities have an extended configuration in solution and will be deposited in same configuration as lying flat on the surface, thus forming a thin and compact coating. Polysaccharides with low charge densities have random coil-like configuration in solution. When deposited, loops are bound on the surface and tails stick out. This leads to a thick and loose coating.

### Microfluidics-assisted techniques

In microfluidics-assisted coating, microfluidics improves streamlined mixing of coating materials and core particles (F. Yang et al., 2021b). Use of microfluidics could moderate the severity of biological membrane-based coatings by compensating with mixing effects.

Sonication (20–80 kHz) for longer time intervals (up to 1 min) may improve loading capacity but at the expense of the integrity and shape of biological membranes. Liu et al. utilized microfluidics sonication (80 kHz) to synthesize EM-coated, cancer CM-coated, and lipid-coated PLGA NPs (∼170 nm) (Figure 4B) (Liu et al., 2019a). Acoustic pressure was maintained higher than the critical compressive stress to rupture and reassemble the plasma membrane, enabling an instant coating process (<30 ms) with coating efficiencies of more than 87%. Applying only hydrodynamic forces associated with microfluidics is insufficient to break the EM for coating, as only 47.3% of NPs had coating without sonication. Furthermore, the presence of sonication improved the uniformity and stability of the coating.

Microfluidics electroporation reduces the damage to CM by reducing the required voltage (Geng and Lu, 2013). Recently, coating Fe_3_O_4_ NPs (∼80 nm) with RBCMs were reported using microfluidics electroporation (Rao et al., 2017). When the mixture of NPs and the RBCM vesicles flowed through the electroporation zone of the microfluidics chip, the electric pulses could promote the entry of NP into the RBCM-vesicle (Figure 4C). High pulse voltage, high pulse duration, and low flow velocity facilitated the coating efficiency. When compared with the traditional coextrusion, it was observed that for the same degree of coating and stability, fewer numbers of CM vesicles were required per NP. In addition, the coating on particles was more complete and even using the new method, which likely contributed to a superior effect on tumor accumulation and tumor inhibition *in vivo* compared to particles coated by coextrusion.

Zhang and coworkers developed a two-stage microfluidics chip to generate monolayer- and bilayer-lipid coatings around PLGA NPs (Figure 4D) (Zhang et al., 2015, 2016). When PLGA and lipid solutions were fed in the first and second stages, respectively, a lipid monolayer formed around the PLGA cores. When the order of input changed as lipid solution in the first stage and PLGA solution in the second stage, a bilayer lipid coating was produced. Here, the intermediate bilayer lipid vesicles formed in the first stage will reassemble on PLGA cores during the second stage. These particles had rigidity different from the monolayer particle; it is more rigid due to the absence of an interfacial water layer between the core and coating (Sun et al., 2015). NPs with a rigid monolayer displayed higher cellular uptake. It was explained with the assistance of a molecular dynamic (MD) simulation on cellular uptake (Figures 4E,F). While the NPs with the rigid monolayer internalized smoothly by wrapping within CM, the NPs with the soft bilayer deformed its coating and trapped on the surface of the cell.

Microfluidics can be used to generate monodisperse particles with polysaccharide coatings (Yang et al., 2021a; Siavashy et al., 2021). Wu et al. used a multi-capillary microfluidics method to obtain alginate-coated PLGA MPs (Figure 4G) (Wu et al., 2013). PLGA in the oil phase and alginate in the water phase flowed through the inner and outer tubes of the coaxial injection tube, respectively, forming oil-in-water emulsion droplets. Then, another coaxial tube with toluene called a “collection tube” was used to generate double emulsion oil-in-water-in-oil droplets. Droplets were collected into a calcium chloride solution to crosslink the alginate shell and dried, producing solid particles. The size of particles, thickness of the shell, and morphology of layers can be controlled by manipulating the fluid flow rates and geometry of capillaries (Li et al., 2018c).

### Others

Apart from the two-step approach of first preparing lipid vesicles and then using them in coating, lipids have also been directly coated on biomaterials (Krishnamurthy et al., 2015). The solvent exchange method, solvent evaporation method, self-assembly, nanoprecipitation, and thin film hydration are some techniques of direct lipid coating on micron- and nano-scale biomaterials (Yang et al., 2012; Huang et al., 2014a; Palange et al., 2014; Ancona et al., 2018; Hasani-Sadrabadi et al., 2018). These methods usually require a vigorous mixing step to break down lipid micelles in the water phase before or during the coating. Yamauchi et al. wrapped cationic liposomes with neutral lipids to improve plasma stability (Yamauchi et al., 2006a). The neutral lipids, namely, egg phosphatidylcholine (EPC) and DSPE-PEG, can be dissolved in aqueous solutions containing over 50% ethanol, while cationic liposomes were only soluble in 100% ethanol. First, cationic liposomes were suspended in a 62.5% ethanol solution that contains dissolved neutral lipids. Then, distilled water was slowly added to the mixture under continuous stirring until the percentage of ethanol was reduced to 5%. This process deposited neutral lipids on the surface of cationic liposomes, generating “wrapped liposomes.” The authors later found that inclusion of DSPE-PEG in the core cationic liposome results in an efficient and ordered wrapping structure (Yamauchi et al., 2006b). The authors suggested that the deposition process was initiated by the PEG–PEG interaction between DSPE-PEG in the core liposome and DSPE-PEG in the surrounding solution. The subsequent EPC assembly around the core was due to the hydrophobic interactions between the acyl chains of EPC and the acyl chains of DSPE-PEG.

Coating with fatty acid membranes which can divide and change shape by external stimulations may allow coacervates to perform cellular-mimetic operations (Sakuma and Imai, 2015). Tang et al. formed a fatty acid-based membrane around RNA–protein coacervates by the single-step addition of aqueous sodium oleate (Dora Tang et al., 2014). Though the oleic acid concentration was below its critical micelle concentration, the coacervate template prompted self-assembly of the membrane by electrostatic interactions. Compartmentalization behaviors were tested with cationic, anionic, and zwitterionic molecules ranging from 0.3–3.3 kDa. Bare coacervates readily compartmentalized all tested molecules. The fatty acid-coated coacervates showed reduced enrichment of anionic compounds, whereas cationic and zwitterionic compounds were bound to the coating. Large molecules were excluded regardless of the charge. In another work, sodium oleate membrane-enclosed coacervate-based artificial cells mimicked cellular uptake behaviors relying on an external electric field (Jing et al., 2019). Bare coacervates compartmentalized different molecules regardless of size, charge, or polarity. Once coated, coacervates only compartmentalized small polar molecules, whereas cationic hydrophobic molecules and large molecules above 600 Da were excluded. Under an electric field of 10 V/cm, the membrane slipped toward the direction of the electric field while maintaining the membrane integrity (Figures 4H,I), allowing large oligonucleotides to cross the membrane. When the electric field was further increased to 20 V/cm, vacuolization occurred and membrane disintegrated allowing all type of molecules to enter the droplet except HRP (40 kDa) (Figures 4J,K).

Recently, *in situ* synthesis of Pd nanosheets directly inside the exosomes through CO-mediated reduction of Pd^2+^ was demonstrated (Figure 4L) (Sancho-Albero et al., 2019a). First, the authors loaded Pd^2+^ ionic precursors into tumor-derived exosomes by co-incubation. Afterward, Pd nanosheets were generated inside exosomes using CO gas as the reducing agent. This hybrid system displayed homotypic binding, and mediated Pd-triggered dealkylation reactions inside cells with about 97% conversion. This mild strategy avoided any EM protein degradation and maintained the integrity of EM. However, the complex operation process and technological barriers need to be addressed.

After surface modification of biomaterials, the unused coating material should be removed because they aggregate with coated particles. General methods for removal of the excess coating material of solid biomaterials are serial centrifugation and dialysis using distilled water or phosphate-buffered saline (PBS) (Li et al., 2018a; Yeo et al., 2018; Xu C. H. et al., 2020; Stolyar et al., 2021). The authors rarely discussed purification of surface-modified biomaterials. They paid more attention to optimizing the process parameters and core to coating material mass ratio to achieve high coating efficiency (Rao et al., 2017; Yang et al., 2021b). For emulsion droplets, authors are cautious about optimizing the mixing ratio of core and shell components to reduce the non-specific interferences of uncoated colloids in the continuous phase (Dewey et al., 2014).

## Influence of the core biomaterial in surface modification

The size, surface composition, texture, and charge of the core biomaterial play a notable role in the selection of the material and technique for surface modification (Table 7). The size of the core largely influences the choice of the technique. Coextrusion, electroporation, passive loading, freeze-thawing, and coprecipitation are generally reported for NPs due to restrictions in the geometry of equipment, permeability of the biological membranes, or nature of the interactions between coating and core (Hauser et al., 2015; Cheng et al., 2018; Sancho-Albero et al., 2019b). The Pickering emulsion method usually involves micron-size liquid droplets to facilitate the interfacial area of nano-size colloids (Song et al., 2016; Crowe and Keating, 2018). Sonication, adsorption, emulsion evaporation, and electrostatic deposition process rarely had size constraints but mainly depend on surface properties of the core (Martínez Gómez et al., 2008; Song et al., 2021). The surface of the core can be preconditioned to offer conjugating/interacting motifs and favorable surface texture to facilitate surface modification. Polymeric, metallic, ceramic, and protein core particles may have inherent chemical motifs such as hydroxyl, carboxyl, thiol, and amine for the chemical conjugation technique (Z. Li et al., 2018d; Kim, Patel and Patel, 2021). Otherwise, a lipid- or polymer-based scaffold can be coated prior to the chemical conjugation process (Stolyar et al., 2021). Rough surface textures facilitate the physical adsorption technique (Bose, Robertson and Bandyopadhyay, 2018). The surface curvature of nanoscale biomaterials (65–340 nm) was reported to have little effect on the coextrusion method (Luk et al., 2014).

**TABLE 7:** Some examples of surface modification techniques of different type of core particles.

Core particle	Core particle size	Core particle charge	Coating material	Surface modification technique	Reference
Iron oxide NPs	5 nm	−63 mV	Tumor EM	Electroporation	Hood, Scott and Wickline, (2014)
Iron oxide NP	35–50 nm	-	Oleic acid	Sonication	Gupta et al. (2019)
Gold NP	45 nm	-	Tumor EM	Freeze-thawing	Sancho-Albero et al. (2019b)
Gold nanorod	46.5 ± 1.2 nm by 19.0 ± 0.7 nm	+41.6 ± 0.6 mV	Serum proteins	Adsorption	Yeo et al. (2017)
Magnetic nanoplates	52 nm	-	Polysaccharide	Coprecipitation	Stolyar et al. (2021)
Zeolitic imidazolate framework	91.4 nm	+30.6 mV	Tumor EM	Microfluidics sonication	Lv et al. (2021)
PCL NP	130 nm	−57 mV	Dextran	Emulsion evaporation	Lemarchand et al. (2006)
Albumin NP	138.7 ± 3.5 nm	−15.7 ± 2.5 mV	Macrophage CM	Coextrusion	Cao et al. (2020)
PLGA NP	140–150 nm	−14 mV	Dendritic CM	Coextrusion	Cheng et al. (2020)
Silica NP	150 ± 11 nm	−10.8 ± 0.2 mV	Tumor EM	Passive loading	Yong et al. (2019)
RNA–protein coacervates	∼2 µm	+4–30 mV	Fatty acid	Electrostatic deposition	Dora Tang et al. (2014)
Polydopamine-coated PLGA MP	3.3 ± 1.8 µm	-	Serum-derived EM	Chemical conjugation	You et al. (2020)
PLGA MP	3–25 µm	−11.8 to −2.4 mV	Protamine	Double emulsion evaporation	Martínez Gómez et al. (2008)
Tannic acid-doped calcium carbonate particles	∼5 µm	-	10 different proteins with different sizes and isoelectric points	Adsorption	Shi et al. (2019)
DEAE-dextran/dsDNA coacervate droplets	5.4 ± 1.9 µm	+15.1 ± 1.7 mV	RBCM	Pickering emulsion	Liu et al. (2020)
Dextran-in-PEG droplets	∼7 µm	-	Anionic lipid vesicles	Pickering emulsion	Dewey et al. (2014)
Lipid MP	5–38 µm	-	Chitosan	Emulsion evaporation	Scalia et al. (2015)
PLGA MP	∼200 µm	-	Alginate	Coaxial electrospray	Choi et al. (2010)
Starch MP	300–750 µm	-	Chitosan	Adsorption	Song et al. (2021)

In the techniques based on electrostatic interactions such as electrostatic deposition and adsorption, the surface charge of the core is important. A precise balance between charges may be required according to the end application. For example, fluidity of the coating is important in T-cell targeting applications to facilitate the immune synapse (Yokosuka and Saito, 2010). Hence, strong binding by strong electrostatic interactions is undesirable in such applications. Most core particles of CM coatings are neutral or negative because positive particles interact strongly with the negative CM, resulting in aggregates of positive NPs and negative CMs (Liu et al., 2021; Wang et al., 2021). CMs have an outside orientation that faces the extracellular environment while an inside orientation that faces the cytosol. The negative cores further facilitate outside-out orientation of the CM coating by electrostatic repulsion (Hu et al., 2013; Luk et al., 2014). The extracellular side of CMs possesses a strong negative charge owing to abundant sialylated moieties. Therefore, the less negatively charged intracellular side of the CM is likely to interact with the negative core.

## Characterization of surface modification

Physicochemical and biological characterizations are required to uncover the effects of coating on material properties and material function. This section discusses some common characterization techniques that would reveal different types of information about the coating.

### Chemical/biochemical identities of the surface modification

Chemical/biochemical identities of the surface medication are important to identify functional capacity of the coating. For coatings with CM, EM, and proteins, SDS-PAGE is used to ensure translocation of surface membrane proteins or binding of protein. SDS-PAGE separates proteins based on their molecular weight. Protein profiles of isolated CM/EM or protein, uncoated particles, and membrane-coated particles will be analyzed to find common markers. A reference protein ladder may use to get a qualitative estimation of the protein identities. To distinguish a specific membrane protein, Western blotting can be performed. Protein quantity of the coating is measured by bicinchoninic acid (BCA) or Bradford assay (Morishita et al., 2017; Jung et al., 2019).

X-ray photoelectron spectroscopy (XPS) and Fourier transform infrared spectroscopy (FTIR) can verify the chemical composition of the coating. XPS measures elemental composition, chemical, and electronic state of the atoms. It is based on the photoelectric effect, and its main components include an X-ray source, vacuum chamber, electron collection lens, and electron energy analyzer. By comparing the XPS spectrum of bare and modified material, the presence of coating can be verified. Quantification of surface-bound elements also can be performed, but it is challenging and rarely used. XPS analysis penetration depth is about 10 nm, limiting its use to thin coatings (Bao et al., 2018). You et al. examined the XPS nitrogen peak at binding energy of 400 eV to confirm surface coating of exosomes on PLGA cores mediated by polydopamine (You et al., 2020). After polydopamine functionalization, a new peak corresponding to amine groups of polydopamine was observed in the XPS spectrum. When EM was incorporated, the peak intensity was intensified, accounting for amine groups of EM. FTIR determines chemical composition by its spectrum that represents the molecular bond absorption and transmission. While XPS is a high energy/short wavelength method that generates a spectrum through excitation of electrons, FTIR is a low energy/high wavelength method that is limited to the material surface and records energy of molecular bonds. Usually, researchers apply both methods in interpreting the mechanism and interactions of coating.

Raman spectroscopy (RS) is a highly sensitive technique for the analysis of molecular structures and compositions even at low concentrations. The Raman spectrum is generated by exciting a sample using a high-intensity laser beam and measuring the Raman effect (energy difference between the incident light and the scattered light due to molecular vibrations). This generates structural fingerprints for different analytes. Surface-enhanced Raman spectroscopy (SERS) enhances the Raman shifts of analytes prepared on gold/silver nano-substrates by plasmon-enhanced excitation and scattering (Bhowmik et al., 2015). Despite the significant improvements in RS techniques, difficulties in reproducibility discourage its use. The acceptable error range for RS is ∼20% (Pérez-Jiménez et al., 2020). Nuclear magnetic resonance (NMR) spectroscopy is a non-invasive quantitative method to identify and characterize chemical structures. The resonant frequency of a selected atomic nucleus under a magnetic field is recorded to find structural peak shifts. NMR is commonly used to verify chemical and structural information of functionalized and purified polysaccharides. Chen and coworkers verified generation of core-shell NPs using the NMR spectrum (Chen et al., 2018). NMR techniques can also be used to study dynamics of lipid-based membranes (Lombardo and Kiselev, 2022).

### Physical characteristics of the coating

**FIGURE 5:**
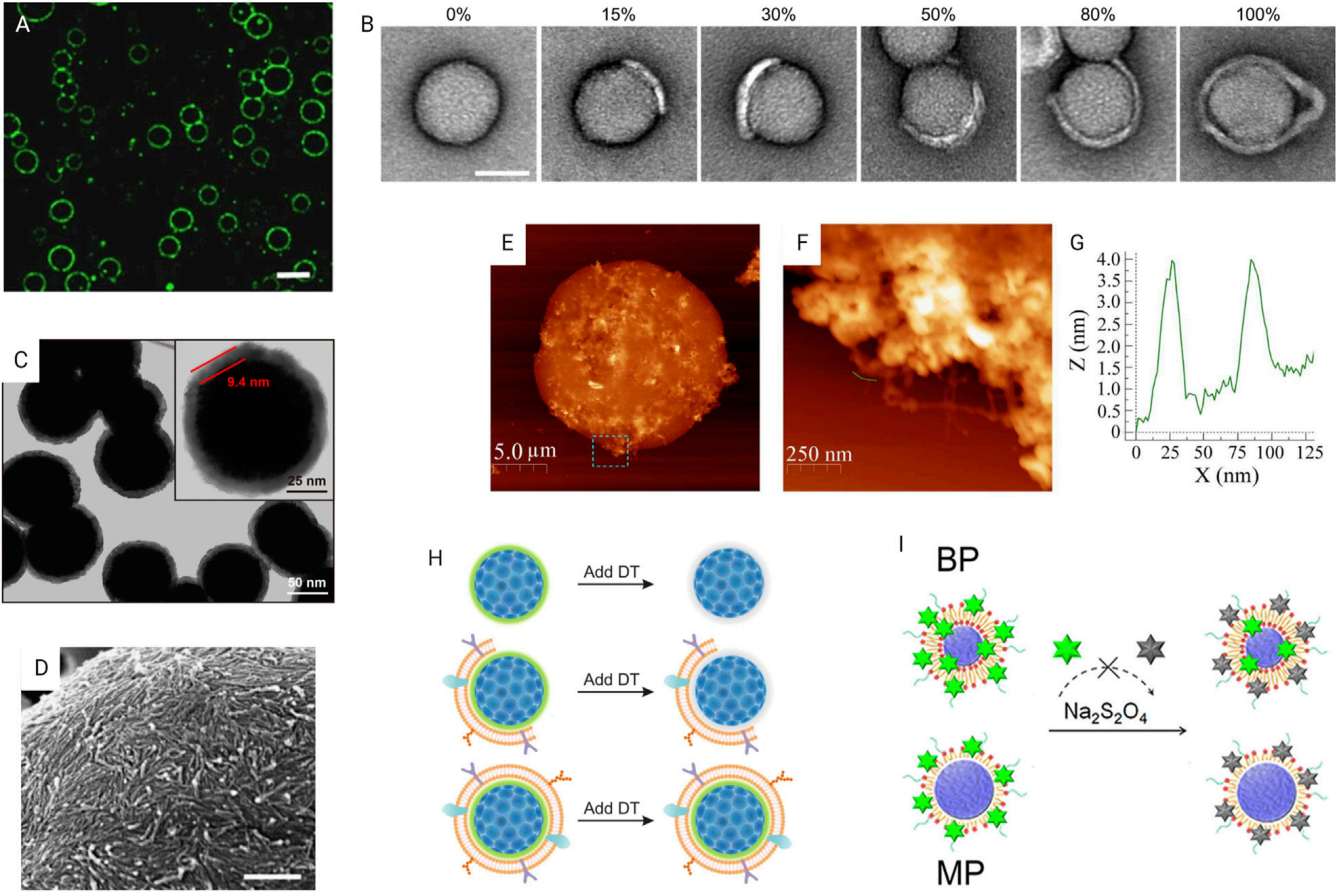
Characterization of surface modification. **(A)** Confocal fluorescence microscopy image of lysozyme fibril-coated w/w emulsion droplets. Scale bar, 20 mm by Song et al. (2016) licensed under CC BY 4.0. **(B)** TEM images of CM-coated NPs with different coating percentages. Scale bar, 50 nm (Liu et al., 2021) licensed under CC BY 4.0. **(C)** TEM image of RBCM-coated NPs with enlarged image showing thickness of the coating; Reprinted with permission from Rao et al. (2017). Copyright 2017 American Chemical Society. **(D)** SEM image showing accumulation of nanofibrils in a self-assemble coating. Scale bar, 500 nm by (Song et al., 2016) licensed under CC BY 4.0. **(E)** AFM image showing the surface texture of a hydrogel-outgrown proteinosome with the **(F)** magnified image of the dashed box area marked in **(E)** showing outgrown hydrogel filaments and **(G)** corresponding height profile measured by AFM to determine filament thickness of 2–4 nm by (Mu et al., 2021) licensed under CC BY 4.0. **(H)** Fluorescence quenching assay of quantifying fully coated particle percentage by Liu et al. (2021) licensed under CC BY 4.0. **(I)** Fluorescence quenching method using NBD-labeled lipid-PLGA NPs to verify the lipid shell structure. Reprinted with permission from Zhang et al. (2015). Copyright 2015 American Chemical Society.

Microscopic techniques can be used to qualitatively assess the surface coating. Confocal fluorescence microscopy is applicable for micron-scale biomaterials. The core and/or shell should be labeled/stained with contrast fluorescent dyes. A clear outline of the coating may be observed depending on the resolution of the microscope (Figure 5A). However, this method is not suitable to verify the completeness of the coating. Even though some studies considered colocalized fluorescence signals of the core and shell of NPs as successful coating (Yong et al., 2019; Xu C. H. et al., 2020), TEM imaging is better for visualizing nanoscale coatings. Liu et al. used TEM images to determine the completeness of the CM coating (Figure 5B) (Liu et al., 2021). The use of TEM images to measure the nano-size thickness of the coating is reasonable and a well-used practice. In a study of RBCM-coated NPs, coating thickness of ∼9.4 nm by TEM image is consistent with reported natural RBCM thickness (5–10 nm) (Figure 5C). In a study validating the TEM approach to sizing nanomaterials, the authors revealed that expanded measurement uncertainty of the TEM approach is 7–20%, depending on the material and image analysis mode (Verleysen et al., 2019). Scanning electron microscopy (SEM) (Figure 5D) and atomic force microscopy (AFM) (Figure 5E) are used to observe the surface morphology of the coating. Although SEM uses an electron beam, AFM uses fluctuations of a contacting mechanical probe to produce details. AFM can also quantify the elasticity (young’s modulus) and dimensional information of the surface (Figures 5E–G).

In a study assessing CM coating completeness, it was found that more than 80% NPs are partially coated regardless of whether the coating method is either coextrusion, sonication, or a combination of them (Liu et al., 2021). A fluorescence quenching assay was developed to calculate the coating percentage. Nitro-2,1,3-benzoxadiazol-4-yl (NBD) loses its fluorescence, when reacted with CM impermeant sodium dithionite (DT). NPs were labeled with NBD prior to the coating. When exposed to DT, fully coated NPs will retain their fluorescence, while partially coated or uncoated NPs progressively reduce their fluorescence (Figure 5H). Authors quantify the proportion of fully coated NPs by measuring the remaining fluorescence. Another group used an aggregation assay based on streptavidin–biotin crosslinking chemistry to assess the completeness of surface coating (Luk et al., 2014). Biotinylated core particles will aggregate or bind with each other in the presence of streptavidin if they are not fully coated.

Zhang and coworkers introduced three techniques to differentiate mono- and bilayer coatings (Zhang et al., 2015). They prepared polymeric NPs with monolayer (MP) and bilayer (BP) of lipid coatings. First, cryo-transmission electron microscope (cryo-TEM) images and electron density across the membrane were analyzed to qualitatively distinguish mono- and bilayers. In the second method, the mass of lipid required for each particle was quantified. Particles were coated using different amounts of lipid, and a minimum amount of lipid that gives the colloidal stability was taken as the mass required for complete coating. As expected, the lipid mass of BP was double that of MP. Finally, a fluorescence quenching method was used. Lipids with NBD-labeled heads were used to coat the NPs. Next, the DT quencher was slowly added to NPs while measuring decrease in fluorescence intensity (Figure 5I). After three drops of DT, fluorescence of MPs was completely quenched, whereas BPs only reduced its fluorescence by half. Six drops of DT completely quenched the BPs’ fluorescence. This NBD quenching method is based on the fact that DT reacts more rapidly with NBD in the outer lipid layer than the inner layer as controlled by dropwise addition.

In CM and EM-based coatings, proper outside-out orientation is important for molecular interactions. In a work with RBCM-coated polymeric particles, the authors confirmed outside-out orientation by labeling NPs with anti-CD47 antibodies specific to the CD47’s extracellular region (Luk et al., 2014). The samples were visualized using the TEM with the assistance of a gold-conjugated secondary antibody. They further confirmed the results by showing the absence of immunostaining with anti-CD47 that specifically targets the intracellular sequence of CD47 used. The same team later used comparatively simple methods to verify the orientation (Luk et al., 2014). The first method quantified the glycan on the particle surface as glycans are asymmetrically distributed on the extracellular side of CMs. An RBCM impermeable enzyme, trypsin, was used to extract glycoproteins. In the second method, sialic acid, a characteristic carbohydrate terminus on RBC glycan, was quantified. The change of charge with sialidase treatment was measured to quantify the surface sialic acid content. Obtained results were similar to those of previous study (outside-out orientation).

In applications such as immunotherapy, fluidity of the surface coating is important for the presentation of signaling molecules to cells (Cheung et al., 2018; Olden et al., 2018). Fluorescence recovery after the photobleaching (FRAP) experiment could be used to characterize fluidity and diffusivity of the coating. Under FRAP experiments, a defined area of fluorescence-labeled coating is photo-bleached instantly, and fluorescence recovery is recorded for usually less than 15 min. Percentage recovery over time can be used to calculate membrane diffusivity. An unbleached, reference region should be used to normalize the acquired fluorescence intensity values as the sample itself bleaches during scanning.

### Indirect assessment of functions of coating

Achieving colloidal stability is one of the common reasons for surface modification of colloidal biomaterials. In usual practice, the colloidal material will be stored in PBS, different percentages of fetal bovine serum (FBS) or blood, and change of the size, surface charge, or surface functionalities will be checked for several days (5–21 days) (Curtis et al., 2018). In general, authors prefer to measure the particle size. Dynamic light scattering is the most popular method of the measuring size and polydispersity index of small biomaterials with a maximum size limit of ≈8 µm. For larger micromaterials, image analysis software-assisted sizing is applicable using optical microscopy images. The surface charge can be obtained using the zeta potential analyzer. Recently, a research team declared that constant agitation during storing is essential to get actual results of stability (Yang et al., 2021b). They observed that regardless of whether the NPs were coated or uncoated, without constant agitation, particles exhibited no change in size or polydispersity. Conversely, with agitation, bare particles showed a significant size increment due to aggregation of poorly coated particles. To mimic the *in vivo* environment in the stability test, constant agitation might be reasonable.

Most of the self-assembled coatings of lipids, CM or EM, were prepared using the vesicles of the coating material. Once assembled on the materials’ surface, vesicles may fuse to form a bilayer or remain as a layer of vesicles. This influences permeability and lateral diffusivity characteristics of the membrane. Pir Cakmak et al. used a calcein-based method to distinguish whether the vesicles are fused in their lipid-based coating (Pir Cakmak, Grigas and Keating, 2019). Calcein is a self-quenching fluorescence dye. When encapsulated at high concentrations in vesicles, it shows a negligible fluorescence, and if vesicles fuse to form a continuous bilayer membrane, vesicles disrupt dramatically increasing fluorescence.

Cellular uptake can be qualitatively assessed using a confocal fluorescence microscope. Flow cytometry is usually used to quantify cellular uptake by fluorescent labeling of core particles. To determine the endocytosis pathways of cellular uptake, the cells was treated with commercially available endocytosis inhibitors prior to the uptake experiment. After *in vitro* evaluations, biodistribution of the particles was monitored for 24–48 h for *in vivo* targeting studies. Animals were injected with fluorescent-labeled particles, and the fluorescence distribution was monitored using an *in vivo* imaging system (IVIS Spectrum). Additionally, the animal was euthanized, and its vital organs, excised for *ex vivo* fluorescence imaging. The fluorescence intensity of *ex vivo* images may be used to quantify bioaccumulation of each organ. Furthermore, in pharmaceutical applications, a new drug release profile after coating should be assessed. It was observed that the release profile tends to be delayed, probably due to reduced drug diffusivity through coating (Zhang et al., 2008; Gao et al., 2016). In a study of EM-coated NPs (∼130 nm), EM coating has not only led to a higher therapeutic effect but also improved the drug diffusion profile by preventing the burst release (Xiong et al., 2019). The *in vitro* release kinetics was evaluated *via* incubating particles in PBS with (out) constant agitation. At preselected time points, the release medium was collected to measure the released drug concentration. The release medium also needs to be completely replaced at this time point to maintain sink conditions. For *in vivo* pharmacokinetic experiments, the blood serum of blood samples collected at different time points was separated by centrifugation and then analyzed by high-performance liquid chromatography (HPLC) or mass spectrometry.

After coating, evaluation on biocompatibility of the new surface should be performed. Biocompatibility includes evaluation on immunotoxicity, hemocompatibility, infection, and tumorigenesis. However, in the research stage, most of the authors limit biosafety evaluation to several tests. In the *in vitro* stage, microscopic examination of cell morphology and cell viability assays such as MTT, CCK-8, or live/dead assay were used to estimate the toxicity. *In vivo* immune assessments include weight measurement of the animals, histology analysis and immunostaining of tissues, and blood examination.

## Summary

The purpose of a surface coating can vary significantly from a biologically inert barrier to a highly immunogenic stimulator. This review summarized the techniques of surface modification of micron- and nano-scale biomaterials using biological membranes and biomolecules. Lipids, fatty acids, and polysaccharides are well-studied surface coating materials with advantages of a wide variety of materials to select, high design flexibility, and high control over the coating process. They were widely used as scaffold materials for further functionalization with proteins and peptides. Surface modification with proteins and peptides allows biomaterials to actively interact with cells *via* receptor signaling and binding. CMs and exosomes are useful coatings because of the rich biological information they carry, despite the difficulty of their comprehensive characterization. However, use of the crude membrane would affect the control over composition of the coating since the physiological state of cells influences the composition of CM and EM; standardized laboratory practices should be implemented to ensure reproducible and reliable results.

Table 8 summarizes advantages and limitations of different surface modification techniques, in the context of biological membrane- and biomolecule-based coatings. Most of the techniques of surface modification such as adsorption, sonication, and self-assembly are common in all the materials discussed. Coextrusion, electroporation, and freeze-thawing were useful for synthesizing CM-, EM-, and lipid-coated NPs. Techniques with electric and thermal processes were mainly used in chemically robust polysaccharides and lipids. In recent years, microfluidics devices have become popular to achieve high throughput and high control in coating (Zhang et al., 2015; Liu et al., 2019a).

**TABLE 8:** Advantages and limitations of different surface modification techniques.

Method	Advantage	Limitation
Coextrusion	High degree of control reproducibility	Clogging
	Preserves orientation of the vesicle	Exosomes prone to aggregate after extrusion
		Preformed vesicles are required
Sonication	Applicable to both micron- and nanosized biomaterials	Low yield of fully coated particles
	High throughput	
	Less loss of material	
Adsorption	Simple	Unstable
	Applicable to both micron- and nanosized biomaterials	Possibility of non-specific interactions
		May require a preconditioned surface
Chemical conjugation	High control over degree of surface modification	Possible denaturing effects
	Applicable to both micron- and nanosized biomaterials	
Pickering emulsion	Simple and fast	Less control over stability
	Preserves dynamic nature of emulsion droplets	High requirement in optimizing mass ratio of cores and coating materials
Electroporation	Simple and fast	Possible low encapsulation yield
		Preformed vesicles are required
Passive loading	Simple	Suitable for NPs
	Non-invasive	Preformed vesicles are required
Freeze-thawing	Preserves integrity, structure, and composition of biological membranes	Suitable for NPs
		Changes the orientation of the coating vesicle
Coprecipitation	Easy scale-up	Harsh chemical process
	Allows control over the particle size	Not suitable for biological membranes
Emulsion evaporation	High encapsulation yield	Less control over polydispersity
	Applicable for both hydrophobic and hydrophilic particles	Requires organic solvents
	No size restrictions	
Coaxial electrospray	No restrictions in hydrophilicity or hydrophobicity of adjacent layers	Low throughput
Electrostatic deposition	Uniform coating	May require a preconditioned surface
		Expensive
Microfluidics-assisted techniques	Allow precise control	Require large number of auxiliary equipment
	High degree of coating by improved mixing	Expensive
		Clogging

Increasingly, the research community is interested in one-step, simple, scalable, and/or universal surface modification strategies. However, significant challenges exist for complete control over the coating process. Complete coating demands precise control of multiple parameters, which are dependent on both the coating and the core materials. Shortcomings in characterization techniques are a major obstacle in the technique optimization process even in the research setup. Restrictions in scaling up and lack of standard quality control measurements are the common factors hindering the commercial use of some of these techniques.

Much knowledge on process parameters is already available by experience, even though theoretical relationships/equations are missing. The establishment of standardized methods to prepare and characterize physical dimensions, integrity, structure, and efficiency of coating would support the translation of coating technologies. Once theoretical relationships are figured out, computational simulations would accelerate optimization of surface modification. Additionally, integration with microfluidics is a promising tool to control the coating efficiency.
